# Thioredoxins as hormone-responsive redox regulators in wheat: transcriptomic and structural insights

**DOI:** 10.3389/fpls.2026.1911123

**Published:** 2026-10-08

**Authors:** Mohammed Hashim Bouzenita, Rana Adnan Tahir, Mengjie Fan, Aisha Shabib Estanbooli, Kamel Chibani

**Affiliations:** 1 Department of Biology, Sultan Qaboos University, Muscat, Oman; 2 School of Life Sciences, University of Essex, Colchester, United Kingdom

**Keywords:** atypical thioredoxins, docking, hormonal regulation, thioredoxins, typical thioredoxins, wheat

## Abstract

Thioredoxins (TRXs) are small central redox proteins involved in stress responses and plant development, but their hormone-dependent regulation remains poorly characterized. In this study, a genome-wide identification of the TRX family in *Triticum aestivum* (Ta) was performed using *Arabidopsis thaliana* TRXs as orthologs. Phylogenetic analysis allowed the identification of a total of 48 TRXs, including 22 typical TRXs, 22 atypical TRXs, and four thioredoxin reductases (TRs). The identified classes in wheat were conserved compared with the *Arabidopsis* genome; however, no extra classes were identified. To assess the regulation of the wheat *TRX* genes under hormonal treatments, including abscisic acid (ABA), ethylene (ET), indole-3-acetic acid (IAA), and methyl jasmonate (MeJA), an *in silico* analysis was performed using the GEO dataset. The transcriptomic analysis and the quantitative reverse transcription PCR (qRT-PCR) validation revealed that the atypical *TRXs* were more responsive to hormones than the typical *TRXs* and *TRs*. Among these atypical TRX members, atypical cysteine/histidine rich thioredoxin (ACHT), chloroplast drought stress protein of 32 kDa (CDSP32), nucleoredoxin (NRX), and TRX-L3 groups were the most hormone-responsive. ABA strongly upregulated the *TaACHT4.2* gene, whereas *TaNRX1.1* responded to IAA and ET. On the other hand, ABA, IAA, ET, and MeJA downregulated *TaCDSP32.1*, *TaNRX1.2*, *TaNRX1.3*, and *ACHT* members. The molecular docking analysis suggested a potential interaction between the selected atypical TRXs and ABA, IAA, and MeJA. The multidomain TRXs, such as TaCDSP32.1 and TaNRX1.1, showed a high affinity for ABA and IAA, whereas TaTRX-L3.1.3 showed a high affinity for MeJA. This study highlights the importance and potential functions of poorly characterized atypical TRXs as hormone-responsive redox regulatory nodes in wheat and provides a basis for future functional validation under hormonal and stress conditions, as well as during plant development.

## 1 Introduction

Wheat (*Triticum aestivum*) growth and development are strongly affected by heat, drought, and oxidative stress, which are regulated by phytohormone-dependent pathways, including abscisic acid (ABA), gibberellic acid (GA), and auxin signaling (Salvi et al., 2021; Panozzo et al., 2025). These pathways interact with thioredoxin and glutaredoxin systems to regulate development, antioxidant defense, metabolism, and cellular redox homeostasis under stress (Considine and Foyer, 2021).Thioredoxins (TRXs) are ubiquitous redox proteins present in all photosynthetic and non-photosynthetic organisms (Sevilla et al., 2023). The thioredoxin family comprises atypical TRXs, typical TRXs, and the thioredoxin reductase groups. In photosynthetic organisms, TRXS are widely localized in all subcellular compartments such as the cytosol, nucleus, chloroplasts, and the mitochondria (Chibani et al., 2021). Atypical TRX groups, such as atypical cysteine–histidine-rich TRX (ACHT), high chlorophyll fluorescence (HCF164), chloroplast drought stress protein of 32 kDa (CDSP32), Like3, c-type, and h-type TRXs, are chloroplastic, whereas Clot and Cysteine–X–X–Serine (CXXS) proteins are cytosolic, and nucleoredoxins (NRXs) are nuclear. The typical thioredoxins include mitochondrial o-types and chloroplastic f-, m-, x-, y-, and z-types, as well as the thioredoxin-like D (TDX). For the thioredoxin reductases (TRs), the NADPH-dependent thioredoxin reductases (NTRs) operate in the cytosol and chloroplast, while the ferredoxin–thioredoxin reductases (FTRs) are present only in the chloroplast (Chibani et al., 2021; Ancín et al., 2022).

TRXs act as redox regulators that scavenge reactive oxygen species (ROS) by reducing oxidized proteins and antioxidant enzymes, thereby maintaining cellular redox balance (Zhang et al., 2017). Typical TRX proteins are characterized by the presence of a conserved Cysteine–Glycine–Proline–Cysteine (CGPC) active site, whereas the atypical group possesses a CXXC active site (Chibani et al., 2021; Souza, 2025). The active site contains two cysteine residues. The first cysteine (N-terminal) is considered the catalytic one, which breaks the disulfide bridge of the target protein, and the second one, named the resolving cysteine (C-terminal), is able to regenerate the reduced state of TRXs by forming a transient intramolecular disulfide bond that is subsequently reduced by TR subgroups (Li et al., 2025; Souza and Vieira-Neto, 2025).

Phytohormones such as ABA, GA, salicylic acid (SA), methyl jasmonate (MeJA), ethylene (ET), cytokinin (CK), and auxin (AUX) are involved in plant development and stress responses (Khan, 2025). Their signaling pathways are closely linked to cellular redox systems, including glutathione, ascorbate, thioredoxins, and glutaredoxins (Noctor et al., 2018). Through thiol-disulfide exchange, TRXs maintain the activity of oxidant-sensitive transcription factors, receptors, and metabolic enzymes involved in hormone and chloroplast signaling, chloroplast biogenesis, seed germination, embryogenesis, and flowering (Rouhier et al., 2015; De Brasi-Velasco et al., 2025).

Despite growing interest in thioredoxin systems and phytohormonal signaling, the regulation of TRXs by phytohormones remains poorly understood. To address this knowledge gap, we performed a comparative genomic analysis to identify the *Triticum aestivum TRXs* based on their homology with well-characterized *Arabidopsis thaliana* TRXs. We then investigated the transcriptional responses of atypical *TRXs*, typical *TRXs*, and *TRs* to ABA, GA, AUX, CK, ET, and MeJA treatments using publicly available transcriptomic datasets from the Gene Expression Omnibus database, originally generated by Qi et al. (2019). The hormones investigated were limited to those included in the experimental conditions used by Qi et al. (2019). Selected hormone-responsive genes were further validated by quantitative reverse transcription PCR (qRT-PCR). In addition, molecular docking was used to predict hormone-binding affinities and potential interaction sites within selected TRX proteins, thereby identifying candidate proteins that may participate in redox-dependent hormonal regulation. This study enhances the functional annotation of the wheat TRX system, identifies hormone-responsive TRXs, and provides a foundation for future investigations into their roles in wheat development and stress responses.

## 2 Materials and methods

### 2.1 Wheat TRX sequence acquisition and bioinformatic analysis

Reference protein sequences of atypical TRXs, typical TRXs, and TRs from *Arabidopsis thaliana* were used to retrieve the corresponding *T. aestivum* orthologs through BLAST searches in the Phytozome v14 database (https://phytozome-next.jgi.doe.gov/) against the *T. aestivum* genome assembly (IWGSC RefSeq v2.2). Orthologous sequences were selected based on query cover, percentage identity, and the presence of conserved catalytic active sites. The active sites of the predicted *T. aestivum* TRXs and TRs were further compared with those of their potential orthologs in *A. thaliana*. The subcellular localization of *T. aestivum* protein sequences was predicted using TargetP 2.0 (https://services.healthtech.dtu.dk/services/TargetP-2.0/) (Emanuelsson et al., 2000), WoLF PSORT (https://wolfpsort.hgc.jp/) (Horton et al., 2007), and PSORT (https://psort.org/) (Nakai and Horton, 1999) databases to verify their predicted subcellular localizations.

### 2.2 Phylogenetic analysis

The *T. aestivum* TRX protein phylogenetic trees were constructed using Molecular Evolutionary Genetics Analysis (MEGA) software version 12 (Kumar et al., 2024). The phylogenetic trees were built using the neighbor-joining (NJ) method with the pairwise deletion option used to handle alignment gaps, and with the Poisson correction model for distance computation. Bootstrap analysis (1,000 replicates) was performed, with branch lengths scaled according to evolutionary distance (Chibani et al., 2021). The TaTRX protein sequences are available in **Supplementary Material S1**.

### 2.3 *In silico* analysis of *TRX* gene expression under hormone treatments and heatmap representation

The *TRX* gene expressions linked to the *T. aestivum* transcriptome under several hormonal treatments (Qi et al., 2019) were acquired using the Gene Expression Omnibus (GEO) database under the accession number GSE103430 (https://www.ncbi.nlm.nih.gov/geo/query/acc.cgi?acc=GSE103430). The SOFT family files containing the expression values and probe annotations based on the GeneChip Wheat Genome Array were manually examined to identify wheat-specific *TRX* candidates based on the annotated associations with known typical and atypical *TRXs* as well as *TRs* previously characterized in *A. thaliana* (Chibani et al., 2021).

The data of identified wheat *TRX* and *TRX* reductase gene candidates were then analyzed for variations in expression as found in the publicly available matrix series file based on an absolute log₂ fold-change threshold of 0.5 (|log₂FC| ≥ 0.5). R software v4.5.1 was used to create a hierarchical heatmap using the heatmap.2 function from the gplots package (R Core Team, 2023). The overall trends and patterns of gene expression observed in the heatmap were computationally verified by applying a combination of Bioconductor packages GEOquery, affy, gcrma, and limma. The verification data were based on the MAS5 normalization of raw GSE103430 data scaled to 100 (sc = 100) and RMA normalization. Statistical significance was determined using the Benjamini–Hochberg false discovery rate (BH-FDR) correction for multiple comparisons. Significant differences were indicated by asterisks: ^*^BH-FDR < 0.05, ^**^BH-FDR < 0.01, and ^***^BH-FDR < 0.001.

### 2.4 *T. aestivum* growth conditions and hormone treatments

Seeds of *T. aestivum* (Roblin cultivar) were sterilized with 70% ethanol for 30 s, treated with 30% sodium hypochlorite for 5 min under gentle shaking, and rinsed 10 times with distilled water to remove residual bleach (Bošnjack et al., 2024). Seeds were dried before the seed germination tests. Seeds were grown in a controlled environment in a CONVIRON ADAPTIS growth cabinet at 25°C under a 16-h light/8-h dark photoperiod with a night temperature of 20°C and 60% relative humidity (RH). According to the developmental requirements of wheat, the plants were watered as needed and fed with NPK 15–15–15 fertilizer on a weekly basis according to the protocol described in Qi et al. (2019).

A minimum of three biological replicates was used for each hormone treatment. At the mid-anthesis stage, the flower heads (spikes) were treated for 24 h with freshly prepared 0.38 mM ABA, 2 mM IAA, and 1 mM MeJA. For ethephon treatment, heads were sealed in plastic bags with 2 mL of 25 mM ethephon (pH = 11). A 10% methanol control was included to account for potential variations induced by methanol rather than hormone exposure. Three biological replicates were used to evaluate plant development and sample condition (Qi et al., 2019).

### 2.5 Quantitative reverse transcription PCR analysis

Total RNA was extracted from wheat spikes at mid-anthesis stage using the NucleoSpin RNA/Protein Kit (Macherey–Nagel GmbH & Co. KG, Düren, Germany), following the manufacturer’s instructions (http://www.mn-net.com/). First-strand cDNA was synthesized from 1 µg of total RNA using the RevertAid Reverse Transcriptase (Fermentas, Vilnius, Lithuania) for downstream gene expression analysis. Primers were designed based on the coding sequence of the identified proteins, taking into account potential overlap among the identified protein sequences. Given the polyploid nature of the wheat genome, each putative *TRX* sequence was blasted against the Phytozome database to identify corresponding copies across the A, B, and D subgenomes. Single nucleotide positions (SNPs) were identified and used to guide primer design. Primer3Plus (https://www.primer3plus.com/index.html) was used to generate the primer sequences. The reference gene and TRX primer sequences are listed in **Supplementary Table 1**. The β-actin reference gene was selected based on the study of Zhou et al. (2022). The primer sequences are provided in **Supplementary Material S2**.

A minimum of three biological replicates was analyzed for each hormonal treatment, and each biological replicate was analyzed in technical triplicate using an IQ Thermal Cycler (Bio-Rad Laboratories, Inc., Hercules, California, USA) with qPCR SYBR Green Master Mix (PCR Biosystems Ltd., London, United Kingdom). The data were normalized using the β-actin reference gene reported by Zhou et al. (2022). Relative expression levels were calculated using the 2^−ΔΔCt^ method (Livak and Schmittgen, 2001). Data were analyzed using Bio-Rad CFX Manager 3.1 software. Differences between each hormonal treatment and the control (untreated samples) were evaluated using Welch’s *t*-test, and the resulting *p*-values were adjusted for multiple comparisons using the BH-FDR correction for multiple comparisons. Significant differences are indicated by asterisks: ^*^BH-FDR < 0.05, ^**^BH-FDR < 0.01, and ^***^BH-FDR < 0.001.

### 2.6 Three-dimensional protein structure predictions

The three-dimensional (3D) structures of the proteins were acquired either by identifying high-confidence homologs available in the Protein Data Bank database (PDB) (https://www.rcsb.org/). Proteins with unavailable or low-confidence entries in official databases were then predicted via two approaches: first, Phyre v2.2 (https://www.sbg.bio.ic.ac.uk/~phyre2/html/page.cgi?id=index), a homology-based protein structure prediction engine (Powell et al., 2025), and subsequently D-I-TASSER (https://aideepmed.com/D-I-TASSER), a deep learning-based iterative threading assembly refinement method (Zheng et al., 2025). Model quality predictions were verified using the ERRAT and PROCHEK tools available through the UCLA-DOE LAB SAVES v6.1 web server (https://saves.mbi.ucla.edu/). The structural results obtained using the two prediction methods were compared to determine the highest quality predicted model for each TRX protein.

### 2.7 Two-dimensional chemical structures of the phytohormones

The 2D chemical structures of the hormones ABA, indole-3-acetic acid (IAA), and MeJA were retrieved for the docking analysis from the PubChem website (https://pubchem.ncbi.nlm.nih.gov/) (Kim, 2021; Kim et al., 2021). The PubChem compound identifiers were Compound identifier (CID) 5280896 for ABA, CID 802 for IAA, and CID 5281929 for MeJA.

### 2.8 Docking analysis

Protein-hormone docking was performed using AutoDock Vina implemented in UCSF ChimeraX software version 1.2.7 on a local machine and facilitated by the PyRx program (Dallakyan and Olson, 2015). The ligand structures corresponding to the hormones were obtained from the ChEBI database, the Chemical Entities of Biological Interest Database (https://www.ebi.ac.uk/chebi/) (Hastings et al., 2015) in the Molecular file (MOL) format. All MOL-formatted ligand structures were energy-minimized and converted to PDB format using the Molecular Operating Environment (MOE) prior to being imported into PyRx for docking analysis. A cutoff value of ≤ − 6.5 kcal/mol, based on blind docking, was used to select proteins with the highest predicted binding affinities. The cutoff values are available in **Supplementary material S3**.

## 3 Results and discussions

### 3.1 Genome-wide identification and phylogenetic analysis of the wheat thioredoxin family

Based on the transcriptomic data obtained from the wheat study by Qi et al. (2019), we conducted a genome-wide identification and characterization of *Triticum aestivum* TaTRX proteins and genes, providing a clear and comprehensive classification of the wheat thioredoxin family, including typical and atypical TRXs, as well as TRs. *Arabidopsis thaliana* AtTRX sequences obtained from the TAIR website were used as queries to identify and confirm the wheat TRX homologues through protein BLAST searches against the phytozome platform v15 database (Goodstein et al., 2012). A total of 22 typical TaTRXs, 22 atypical TaTRXs, and four thioredoxin reductases (TaTRs) were identified in wheat. TaTRXs were distributed among six well-defined typical classes, seven atypical classes, and two TaTR classes (**Table 1**). The typical TaTRXs comprised the f, m, y, o, h, and TDX types. The atypical TaTRXs included ACHT, CDSP32, Like3 (L3), Clot, CXXS, TRX-c type, and NRX types (**Figure 1**). Subcellular localization predictions obtained using PSORT, WoLF PSORT, and TargetP 2.0 confirmed the localization patterns reported for typical and atypical TRXs in *P. trichocarpa* and *A. thaliana* (Chibani et al., 2009, 2021). Typical f, m, y, and TDX types, as well as the atypical CDSP32, ACHT, TRX-Like3, and the TRX c-type, were predicted to be chloroplastic. Typical o-type TaTRXs were predicted to be localized in the mitochondria. Typical TaTRX-h type and atypical CXXS and CLOT proteins were predicted to be localized in the cytosol. In addition, atypical TaTRXs include a single class of NRXs localized in the nucleus.

**Table 1:** Classification and major features of the *Triticum aestivum* thioredoxin (TaTRX) family and its *Arabidopsis thaliana* orthologs.

TRX group^a^	*Triticum aestivum* (Ta)						*Arabidopsis thaliana* (At)			
	Class	Gene name^b^	Gene models^c^	Protein ID^d^	Active site^e^	Subcellular localization^f^	Gene name^g^	Gene models^h^	Protein ID^i^	Active site^j^
Typical TRX	f	*TaTRX-f1.1*	*Ta.872.2.S1_at*	Traes_3AL_6420E8378.1	WCGPC	P	*AtTRX-f1*	At3g02730	Q9XFH8	WCGPC
		*TaTRX-f1.2*	*Ta.872.2.S1_x_at*	Traes_3B_3C949D318.1	WCGPC	P	*AtTRX-f1*			
		*TaTRX-f1.3*	*Ta.872.3.S1_at*	Traes_3DL_07178E28A.1	WCGPC	P	*AtTRX-f1*			
		*TaTRX-f2*	*Ta.12945.1.S1_at*	Traes_3B_3C949D318.1	WCGPC	P	*AtTRX-f2*	At5g16400	Q9XFH9	WCGPC
	h	*TaTRX-h1.1*	*Ta.12899.1.A1_at*	Traes_1BL_B1C10EA93.1	WCGPC	C	*AtTRX-h1*	At3g51030	P29448	WCGPC
		*TaTRX-h1.2*	*Ta.24466.1.S1_x_at*	Traes_1DL_DF3CA9CCC.2	WCGPC	C				
		*TaTRX-h1.3*	*Ta.30730.1.S1_at*	Traes_1AL_11FF51731.1	WCGPC	C				
		*TaTRX-h1.4*	*Ta.13723.1.S1_at*	Traes_1BL_AF4C87032.1	WCGPC	C				
		*TaTRX-h1.5*	*Ta.1251.1.S1_at*	Traes_2AL_0ADBF3D27.1	WCGPC	C				
		*TaTRX-h1.6*	*Ta.26879.1.A1_at*	Traes_2BS_9C3ACD499.1	WCGPC	C				
		*TaTRX-h2.1*	*Ta.20239.1.S1_a_at*	Traes_5DL_27BC0CE09.1	WCGPC	C	*AtTRX-h2*	At5g39950	Q38879	WCGPC
		*TaTRX-h2.2*	*Ta.8145.1.S1_at*	Traes_5BL_C6D32C625.1	WCGPC	C	*AtTRX-h2*			
		*TaTRX-h9*	*Ta.4367.1.S1_at*	Traes_1DS_B39E8C867.1	WCGPC	P/M	*AtTRX-h9*	At3g08710	Q9C9Y6	WCGPC
	m	*TaTRX-m1.1*	*Ta.136.1.S1_at*	Traes_2BL_1579D2408.1	WCGPC	P	*AtTRX-m1*	At1g03680	O48737	WCGPC
		*TaTRX-m1.2*	*Ta.14710.1.S1_at*	Traes_2DL_62BDE92C31.2	WCGPC	P				
		*TaTRX-m2*	*Ta.26653.1.A1_at*	Traes_5BS_B72CD04F2.1	WCGPC	P	*AtTRX-m2*	At4g03520	Q9SEU8	WCGPC
	o	*TaTRX-o1*	*Ta.13167.1.S1_at*	Traes_7AL_5AD2BD98D.1	WCGPC	M	*AtTRX-o1*	At2g35010	O64764	WCGPC
		*TaTRX-o2*	*Ta.20864.1.S1_at*	Traes_7BL_BF777344B.1	WCGPC	M	*AtTRX-o2*	At1g31020	Q93VQ9	WCGPC
	TDX	*TaTDX1*	*Ta.6618.2.S1_a_at*	Traes_5DL_CAF80DFD8.2	WCGPC	P	*AtTDX*	At3g17880	Q8VWG7	WCGPC
		*TaTDX2*	*Ta.10647.1.A1_at*	Traes_5AL_097E0BB13.1	WCGPC	P	*AtTDX*			
	y	*TaTRX-y1.1*	*Ta.11839.1.S1_a_at*	Traes_3B_4FCE14A4A.3	WCGPC	P	*AtTRX-y1*	At1g76760	Q6NPF9	WCGPC
		*TaTRX-y1.2*	*Ta.11839.1.S1_x_at*	Traes_3DL_750FC4166.3	WCGPC	P	*AtTRX-y1*			
Atypical TRX	ACHT	*TaACHT2.1*	*Ta.6434.1.S1_at*	Traes_1DS_366988061.2	WCGSC	P	*AtACHT2*	At4g29670	Q8LCT3	WCASC
		*TaACHT2.2*	*Ta.6434.2.S1_at*	Traes_1AS_37383FD3C.1	WCGSC	P	*AtACHT2*			
		*TaACHT2.3*	*Ta.6434.2.S1_a_at*	Traes_1BS_C19C8A810.2	WCGSC	P	*AtACHT2*			
		*TaACHT4.1*	*Ta.7069.1.S1_at*	Traes_4AS_B1D4048C4.1	GCGGC	P	*AtACHT4*	At1g08570	A8MQQ7	GCGGC
		*TaACHT4.2*	*Ta.19627.1.S1_at*	Traes_2DS_5A8D0F48E.1	GCGGC	P	*AtACHT4*			
		*TaACHT6.1*	*Ta.628.1.S1_x_at*	Traes_2AL_63BE91CB7.1	SCGSC	P	*AtACHT6*	At1g07700	Q9C5C5	ACGSC
		*TaACHT6.2*	*Ta.628.2.A1_at*	Traes_2DL_FFF62E69D.1	SCGSC	P	*AtACHT6*			
		*TaACHT6.3*	*Ta.628.3.S1_a_at*	Traes_2BL_D82F44AF2.1	SCGSC	P	*AtACHT6*			
		*TaACHT6.4*	*Ta.628.3.S1_x_at*	Traes_2BL_D82F44AF2.1	SCGSC	P	*AtACHT6*			
	CDSP32	*TaCDSP32.1*	*Ta.2407.1.S1_a_at*	Traes_6DL_C6E63ED1C.1	HCGPC	P	*AtCDSP32*	At1g76080	Q9SGS4	HCGPC
		*TaCDSP32.2*	*Ta.2407.2.A1_at*	Traes_6AL_30768AD47.1	HCGPC	P	*AtCDSP32*			
	Clot	*TaClot*	*TaAffx.121102.3.S1_at*	Traes_7DS_CA226F6E9.1	WCPDC	C	*AtClot*	At5g42850	Q9FMN4	WCPDC
	CXXS	*TaCXXS1.1*	*Ta.3214.1.S1_at*	Traes_2AL_CADDB1089.1	WCVTS	C	*AtCSSXS1*	At1g11530	Q8LDI5	WCIPS
		*TaCXXS1.2*	*Ta.3214.1.S1_x_at*	Traes_2DL_768C1252D.1	WCVTS	C	*AtCSSXS1*			
	c	*TaTRX-c3*	*Ta.13751.1.S1_at*	Traes_3B_2A134A49A.1	VCNLC	P	*AtTRX-c3*	At1g52590	Q9SSR1	VCNLC
	Like3 (L3)	*TaTRX-L3.1.1*	*Ta.13007.1.S1_at*	Traes_6BL_69E800C48.1	WCRKC	P	*AtTRX-L3.1*	At5g06690	Q9FG36	WCRKC
		*TaTRX-L3.1.2*	*Ta.15719.1.S1_at*	Traes_6DL_3D072DB07.1	WCRKC	P	*AtTRX-L3.1*			
		*TaTRX-L3.1.3*	*Ta.7950.1.A1_at*	Traes_6AL_768707567.2	WCRKC	P	*AtTRX-L3.1*			
		*TaTRX-L3.3*	*Ta.15477.1.S1_at*	Traes_2BL_335F9A703.1	WCRKC	P	*AtTRX-L3.3*	At3g53220	Q8LCH9	WCRKC
	NRX	*TaNRX1.1*	*Ta.26561.1.A1_at*	Traes_2DL_28DFAC79D.2	WCGPC/WCPPC	N/C	*AtNRX1*	At1g60420	O80763	WCGPC/WCPPC
		*TaNRX1.2*	*Ta.5649.1.S1_at*	Traes_2AL_D80447132.1	WCGPC/WCPPC	N/C	*AtNRX1*			
		*TaNRX1.3*	*Ta.5649.1.S1_x_at*	Traes_2BL_34819D129.1	WCGPC/WCPPC	N/C	*AtNRX1*			
Thioredoxin reductase (TR)	NTR	*TaNTRA*	*Ta.22568.1.S1_at*	Traes_6DL_3945FC9B7.2	CAVC	C	*AtNTRA*	At2g17420	Q39242	CAVC
		*TaNTRC*	*Ta.6532.1.S1_at*	Traes_2BS_DEC12328A.2	ACAIC/TCGPC	P	*AtNTRC*	At2g41680	O22229	ACAIC/TCGPC
	FTR	*TaFTRA2*	*Ta.23273.1.S1_at*	Traes_6DL_1B50CCE2A.2	EAEHAPK	P	*AtFTRA2*	At5g08410	Q8LBP6	RCDIAVK
		*TaFTRB*	*Ta.12788.2.A1_at*	Traes_6DS_8F0F39831.6	WNCPC	P	*AtFTRB*	At2g04700	Q9SJ89	WNCPC

The *Triticum aestivum* protein sequences are available and provided in **Supplementary Material S1**.

*C*, cytosol; *M*, mitochondria; *N*, nucleus; *S*, secreted; *P*, plastid.

^a^ Symbols of the TRX group are used in the clustering heatmap shown in **Figure 1**.

^b^ The gene names come from our annotation.

^c^ Gene models come from the Phytozome website (https://phytozome-next.jgi.doe.gov/).

^d^ Protein IDs come from the Phytozome website (https://phytozome-next.jgi.doe.gov/).

^e^ The active site was verified using the Phytozome and UniProt (https://www.uniprot.org/) websites.

^f^ Subcellular localization is based on three prediction programs: PSORT (https://psort.org/), TargetP 2.0 (https://services.healthtech.dtu.dk/services/TargetP-2.0/), and WoLF SPORT (https://wolfpsort.hgc.jp/).

^g^ The gene names were obtained from the TAIR website (https://www.arabidopsis.org/).

^h^ The gene models were obtained from the TAIR website (https://www.arabidopsis.org/).

^i^ Protein IDs are available at the UNIPROT website (https://www.uniprot.org/).

^j^ Protein active sites are available at the UNIPROT website (https://www.uniprot.org/).

**Figure 1:**
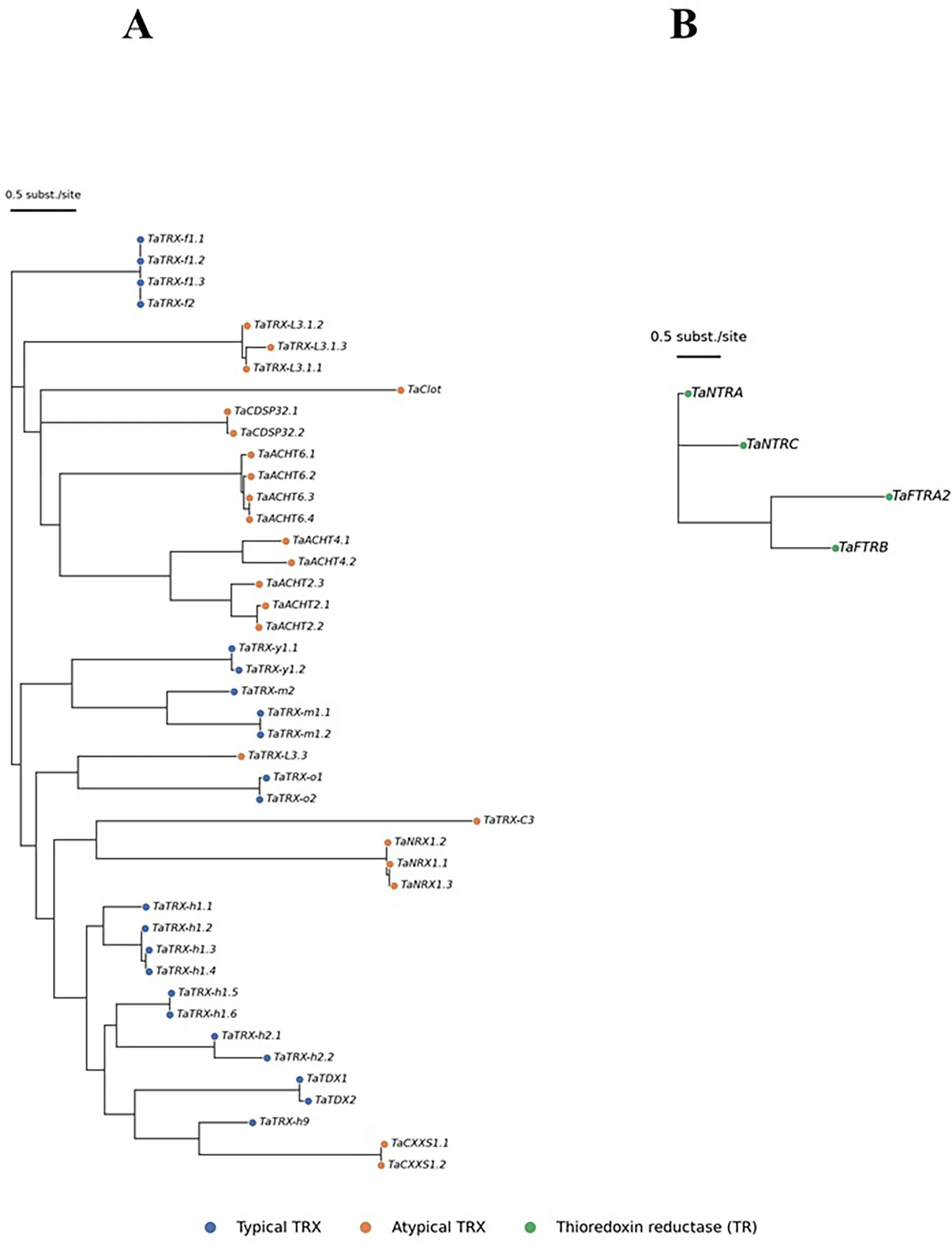
Phylogenetic tree of *Triticum aestivum* thioredoxin family. **(A)** Typical and atypical TRXs. **(B)** Thioredoxin reductase (TRs). The tree was constructed using MEGA4 software v.12. For clarity, *T. aestivum* was abbreviated to Ta, and the protein names have been shortened.

The wheat genome harbors the same major thioredoxin types as *A. thaliana*, including typical and atypical TRXs, as well as the TRs. This conserved classification was consistent with the comprehensive analysis reported by Chibani et al. (2021), which identified the same TRX classes. The limited genome-wide study focusing on typical TRXs by Zhou et al. (2022) confirmed the presence of all major *Arabidopsis* typical TRXs in wheat. The large number of typical TRXs was due to duplication events, which reflect the impact of polyploidization and large-scale genome rearrangements in hexaploids leading to the retention of multiple homologous *T. aestivum* TRX copies. These findings indicate that the core architecture of the thioredoxin system has been evolutionarily conserved between the monocotyledon and dicotyledon species. The differences between wheat and *Arabidopsis* arise from gene duplication and polyploidy-driven expansion rather than the emergence of new typical or atypical TRXs or TRs (Geigenberger et al., 2017; Zhou et al., 2022). In wheat, not all duplicated genes are preserved equally. In some *TRX* classes, certain copies have been lost or are unequally retained, whereas other TaTRXs occur as groups of three related copies, reflecting the polyploid origin of wheat. This suggests a gradual reorganization of the genome after polyploidization (Zhou et al., 2022).

### 3.2 Conservation of catalytic motifs and structural stability

Despite extensive duplication in wheat, TaTRXs maintain a highly conserved redox center similar to that of *A*. *thaliana*, characterized by the canonical tryptophan–cysteine–glycine–proline–cysteine (WCGPC) signature. Most atypical TRXs possess conserved active redox centers. However, the Triticum aestivum atypical Cys-His-rich thioredoxin (TaACHT)6 class displays an serine–cysteine–glycine–serine–cysteine (SCGSC) motif instead of the ACGSC motif observed in *A. thaliana*, while the CXXS class possesses a WCVTS motif instead of the WCIPS signature. In addition, TaFTRA.2 exhibits an EAEHAPK motif instead of the RCDIAVK motif found in *A. thaliana* (**Table 1**). This specific variation may reflect functional diversification within specific atypical TRX-c and TR classes in wheat. Chibani et al. (2021) demonstrated that even in *Arabidopsis*, sequence variations rarely affect the catalytic residues. Wheat TRXs conserve the core structural domains despite extensive duplication (Zhou et al., 2022). These findings suggest that evolutionary diversification in wheat involves regulatory regions rather than the protein core. The functional diversification of wheat TRXs was probably driven by differences in expression patterns, interaction networks, and regulatory responsiveness rather than by changes in enzymatic mechanisms.

### 3.3 Transcriptional modulation of wheat *TaTRX* genes in response to phytohormones

To assess the regulation of typical and atypical *TaTRX* and *TaTR* genes under ABA, MeJA, IAA (Auxin), CK, GA, ET, and SA treatments, we analyzed the Affymetrix dataset from Qi et al. (2019), obtained from the GEO database under the accession number GSE103430. The hormones investigated were limited to those included in the experimental conditions used by Qi et al. (2019). A hierarchical clustering analysis of wheat-related *TaTRX* genes was performed and visualized in a heatmap showing a log_2_ fold change (log_2_ FC) (**Figure 2**) and summarized in **Table 2**. The upregulated and downregulated genes were confirmed by qRT-PCR. From **Figure 1** and the summary represented in **Table 2**, the hormones mainly affected the typical and atypical *TaTRX* genes, whereas *TaTR* appears not to be significantly regulated. Among the hormones tested, ABA showed the strongest stimulatory effect, whereas SA and GA did not induce any significant detectable regulation.

**Figure 2:**
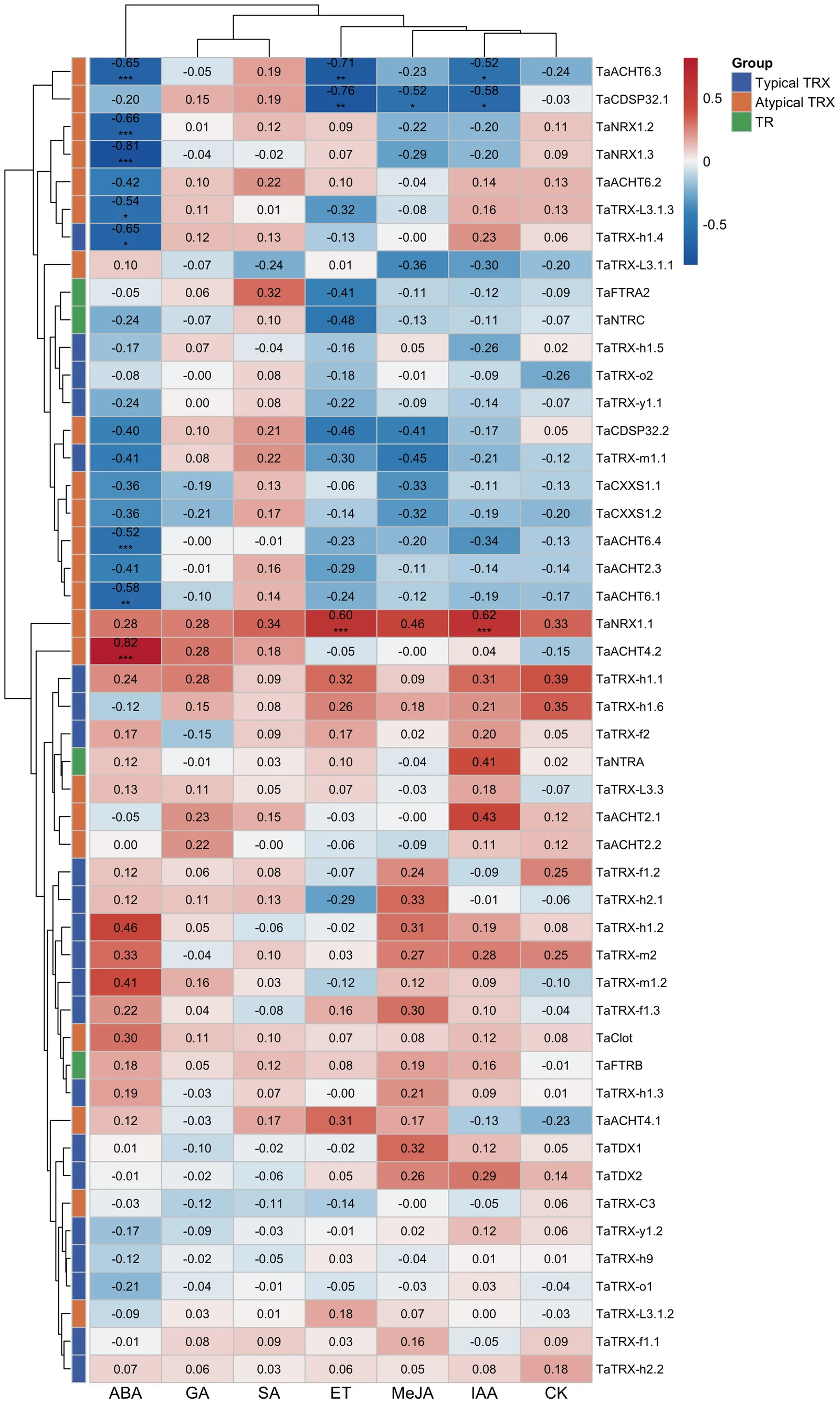
Hierarchical clustering and heatmap representation of the regulation of the typical and atypical *TRXs* and thioredoxin reductase (*TRs*) under hormonal treatments: abscisic acid (ABA), ethylene (ET), indole-3-acetic acid (IAA), and methyl jasmonate (MeJA). The heatmap was constructed using R software v4.5.1. The data in the heatmap represent log_2_ fold changes. Statistical significance was determined using the Benjamini–Hochberg false discovery rate (BH-FDR) correction for multiple comparisons. Significant differences are indicated by asterisks: ^*^BH-FDR < 0.05, ^**^BH-FDR < 0.01, and ^***^BH-FDR < 0.001.

**Table 2:** Hormone-responsive expression patterns of wheat thioredoxin genes.

			Hormones			
TRX group	Class	Gene name	ABA	ET	MeJA	IAA
Typical TRX	h	*TaTRXh1.4*	-0.6			
Atypical TRX	ACHT	*TaACHT4.2*	0.8			
		*TaACHT6.1*	-0.5			
		*TaACHT6.3*	-0.6	-0.7		-0.5
		*TaACHT6.4*	-0.5			
	CDSP32	*TaCDSP32.1*		-0.7	-0.5	-0.5
	Like3	*TaTRX-L3.1.3*	-0.5			
	NRX	*TaNRX1.1*		0.6		0.6
		*TaNRX1.2*	-0.6			
		*TaNRX1.3*	-0.8			

The values are log_2_ fold-change values (log_2_ FC). A cutoff value of ± 0.5 was applied to differentiate upregulated genes represented in red from downregulated genes in blue. Log_2_ fold changes are the same as those represented in the hierarchical heatmap in **Figure 1**.

*ACHT*, atypical Cys/His-rich thioredoxin; *ABA*, abscisic acid; *CDSP32*, chloroplast drought-induced stress protein of 32 kDa; *ET*, ethylene; *IAA*, indole acetic acid; *MeJA*, methyl jasmonate; *NRX*, nucleoredoxin.

#### 3.3.1 Selective upregulation of *TaTRX* genes under hormonal treatments

The hierarchical clustering of the heatmap presented in **Figure 1** separated the expression pattern of the *TaTRX* and *TaTR* genes under different hormonal treatments into two major clusters. The first cluster grouped the upregulated *TaTRX* genes, whereas the second cluster contained the downregulated genes. Among the upregulated genes, only the atypical nucleoredoxin *TaNRX1.1* gene showed significant upregulation under ethylene and IAA treatments, whereas the chloroplastic *TaACHT4.2* gene was significantly upregulated in response to ABA. In contrast, typical *TaTRXs* and *TaTRs* were not significantly upregulated in response to any hormonal treatments (**Table 2**).

The expression patterns of *TaNRX1.1* and *TaACHT4.2*, observed in the transcriptomic analysis, were confirmed by qRT-PCR. *TaNRX1.1* was upregulated under ET and IAA treatments and not under ABA, MeJA, CK, GA, and SA treatments (**Figure 3A**), and *TaACHT4.2* was upregulated only under ABA treatment (**Figure 3B**). In contrast, the weak upregulation of the other chloroplast and mitochondrial *TaTRX* genes suggests that the redox balance remains stable, which may help to preserve essential metabolic activities during photosynthesis and respiration.

**Figure 3:**
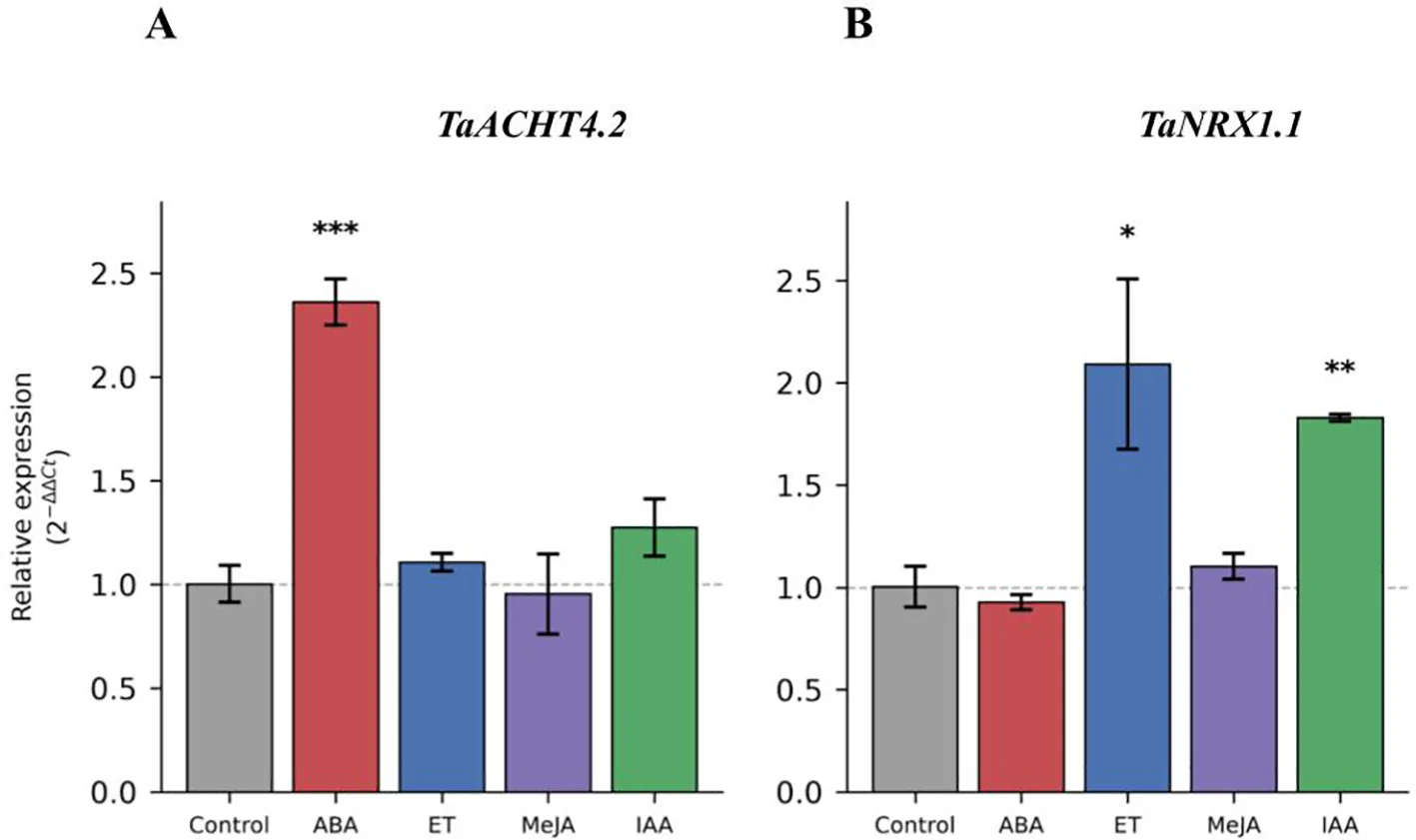
qRT-PCR expression analysis of upregulated *TaTRX* genes under hormonal treatments. qRT-PCR analysis of **(A)** *TaACHT4.2* and **(B)** *TaNRX1.1* under hormonal treatments: abscisic acid (ABA), ethylene (ET), indole-3-acetic acid (IAA), and methyl jasmonate (MeJA). Standard deviations are indicated by error bars (means ± SD; *n* = 3). Differences between each hormonal treatment and the control (untreated samples) were evaluated using Welch’s *t*-test, and the resulting *p*-values were adjusted for multiple comparisons using the Benjamini–Hochberg false discovery rate (BH-FDR) correction for multiple comparisons. Significant differences are indicated by asterisks: ^*^BH-FDR < 0.05, ^**^BH-FDR < 0.01, and ^***^BH-FDR < 0.001.

Plant hormones regulate cellular responses through signaling pathways within the cell that involve hormone perception and the modulation of gene expression (Zhang et al., 2023). *TaNRX1.1* upregulation reinforces the finding that hormone-responsive redox regulation occurs at the nuclear level, as ethylene and auxin signaling pathways converge on the activation of nuclear transcription factors (Delorme-Hinoux et al., 2016). Auxin signaling occurs through the nuclear TIR1/AFB receptor, which promotes the degradation of AUX/IAA repressors, thereby activating auxin response factors (ARFs), which function as nuclear transcription regulators of gene expression (Yu et al., 2022). However, ethylene signaling results in the activation of the *EIN3*/*EIL* and *ERF* transcription factors (Wang et al., 2025). Additionally, the specific upregulation of the *TaACHTA4.2* gene under ABA suggests an ABA-mediated redox regulatory role in the chloroplast. ABA is a key regulator of abiotic stresses such as drought and osmotic stress (Dangoor et al., 2009; Considine and Foyer, 2021). As atypical *TRXs* are known to be hormonal redox sensors, *TaACHT4.2* may contribute to chloroplast transcriptional reprogramming to maintain a redox balance under ABA treatment.

#### 3.3.2 Differential downregulation of *TaTRX* genes under hormonal treatments

ABA treatment downregulated a broader set of genes encoding atypical nuclear and chloroplast proteins as well as a typical cytosolic protein, namely TaNRX1.2, TaNRX1.3, TaACHT6.1, TaACHT6.4, and TRX-h1.4, respectively (**Figure 2**). The expression patterns were confirmed by qRT-PCR, which showed significant repression of all five genes under ABA treatment, consistent with the heatmap results (**Figure 4B**). This transcriptional pattern suggests that ABA exerts a suppressive effect on genes associated with cellular redox regulation across different subcellular compartments such as the nucleus, cytosol, and chloroplast. In *Solanum lycopersicum*, the nuclear gene *SlNRX1* supports antioxidant and stress-responsive transcription positively, rather than acting simply as a negative regulator of antioxidant responses (Cha et al., 2022). The marked downregulation of *TaNRX1.2* and *TaNRX1.3* by ABA therefore likely reflects a reconfiguration of nuclear redox signaling during ABA responses. Likewise, the downregulation of *TaACHT6.1* and *TaACHT6.4* suggested that ABA also affects chloroplast redox homeostasis. This interpretation is biologically consistent because ABA biosynthesis originates from carotenoid precursors in plastids, whereas ABA-responsive transcription occurs in the nucleus, and ABI4 has been identified as an important link between plastid retrograde signaling and nuclear gene expression (Mo et al., 2024). The repression of the cytosolic *TaTRX-h1.4* by ABA is consistent with the involvement of h-type TRXs in seed development, germination, and seedling establishment (Fuchs et al., 2024; Nascimento et al., 2025). Such repression may contribute to the maintenance of an ABA-favored cytosolic environment that restrains germination-associated processes and growth while supporting seed maturation and stress preparedness (Hägglund et al., 2016). These recent findings suggest that *TaTRX-h1.4* may not be restricted to germination but may also participate in transcriptional reprogramming in reproductive tissues, potentially influencing the redox environment required for seed maturation.

**Figure 4:**
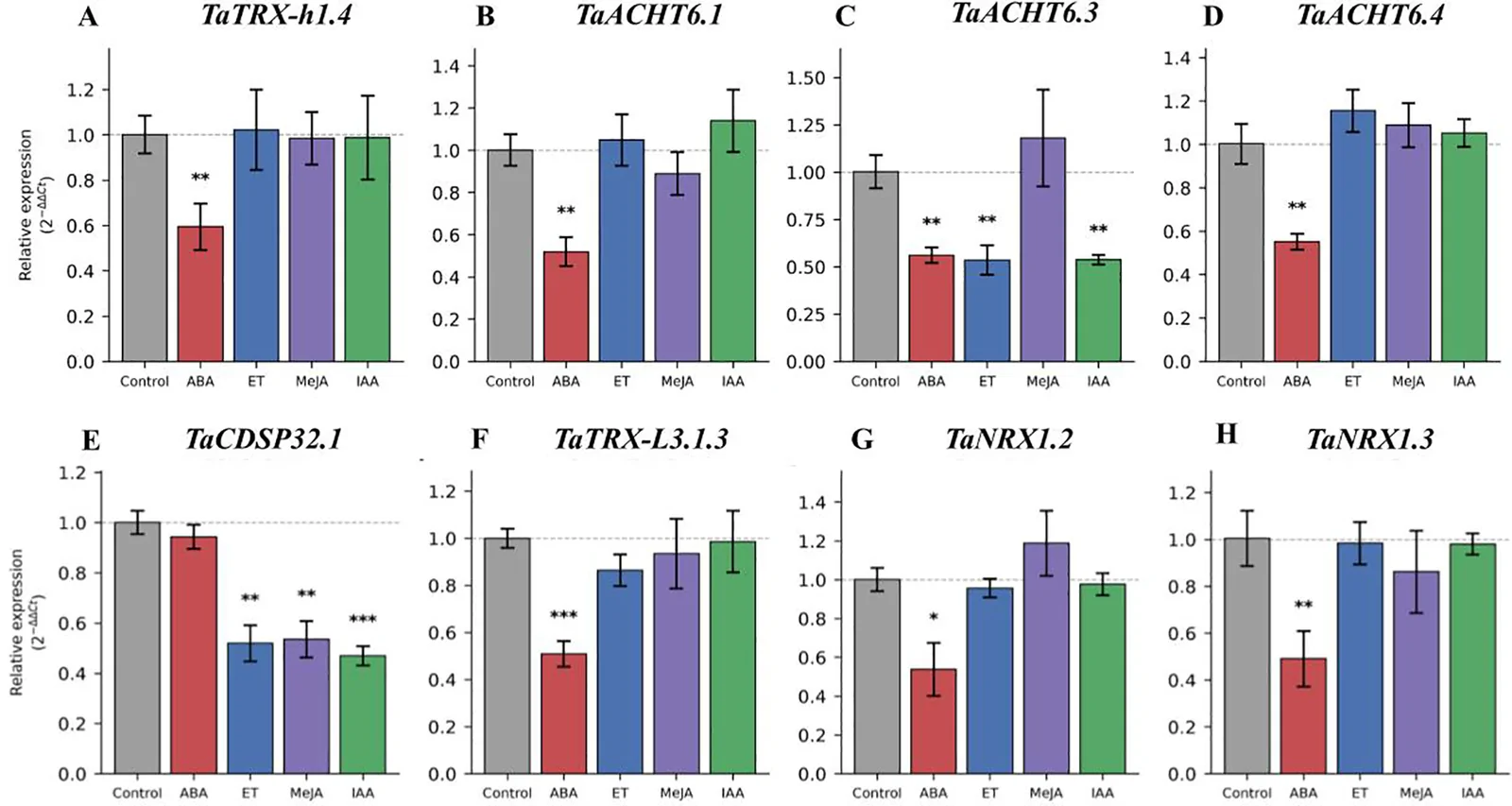
qRT-PCR expression analysis of downregulated *TaTRX* genes under hormonal treatments. qRT-PCR analysis of **(A)** *TaTRX-h1.3*, (**B**) *TaACHT6.1*, (**C**) *TaACHT6.3*, (**D**) *TaACHT6.4*, (**E**) *TaCDSPS32.1*, (**F**) *TaTRX-L3.1.3*, (**G**) *TaNRX1.2*, and (**H**) *TaNRX1.3* under hormonal treatments: abscisic acid (ABA), ethylene (ET), indole-3-acetic acid (IAA), and methyl jasmonate (MeJA). Standard deviations are indicated by error bars (means ± SD; *n* = 3). Differences between each hormonal treatment and the control (untreated samples) were evaluated using Welch’s *t*-test, and the resulting *p*-values were adjusted for multiple comparisons using the Benjamini–Hochberg false discovery rate (BH-FDR) correction for multiple comparisons. Significant differences are indicated by asterisks: ^*^BH-FDR < 0.05, ^**^BH-FDR < 0.01, and ^***^BH-FDR < 0.001.

In contrast, ethylene treatment downregulated a narrower set of genes, including *TaCDSP32.1* and *TaACHT6.3.* The downregulation patterns of *TaCDSP32.*1 and *TaACHT6.3*, observed in the transcriptomic analysis, were confirmed by qRT-PCR. This coordinated repression suggests a specific effect of ethylene signaling on the redox regulatory network. The repressed genes include those encoding CDSP32 and ACHTs, two classes of atypical TRXs involved in oxidative-stress responses, chloroplast redox homeostasis, and the redox regulation of photosynthesis-related enzymes (Rey et al., 2024). Interestingly, *TaCDSP32.1* was not only repressed under ethylene treatment but also under MeJA and IAA treatments, as shown in the heatmap (**Figure 2**; **Table 2**), and confirmed by qRT-PCR (**Figure 4**). This specific pattern indicates that *TaCDSP32.1* may represent a common hormonal target shared by ethylene, jasmonate, and auxin signaling. *TaACHT6.3* was also repressed under ABA, ethylene, and auxin treatments. The downregulation of these plastid-associated genes may reflect hormonal adjustments of redox homeostasis during floral development and the early stages of seed formation. Ethylene is involved in flower development, senescence, fruit ripening, and stress responses (Iqbal et al., 2017; Wang et al., 2025), whereas MeJA and IAA are important regulators of reproductive development and stress- or growth-related signaling (Pandey et al., 2025; Vanneste et al., 2025).

### 3.4 Molecular docking supports hormonal responsiveness of thioredoxins

After identifying a set of atypical TRX-responsive genes in response to hormones, docking experiments were performed to further explore whether these specific gene regulations could be associated with interactions between the proteins and the phytohormones of interest at the post-transcriptional level. To assess this hypothesis, molecular docking was performed between the proteins (TaTRX-L3.1.3, TaCDSP32.1, TaACHT4.2, and TaNRX1.1) encoded by the genes that were significantly repressed or stimulated by the identified hormones (ABA, IAA, MeJA). The results highlighted in this section include only the proteins that showed the highest affinity for the hormones.

#### 3.4.1 3D structure prediction of atypical thioredoxin proteins

The predicted 3D structures of the TaTRX-L3.1.3, TaCDSP32.1, TaACHT4.2, and TaNRX1.1 were represented in **Figure 5**. However, the predicted 3D structures of the TaACHT6 members and TaTX-h1.3 3D were not represented due to the low quality of the structures and the poor affinity of the proteins for the hormones (**Supplementary material S3**). TaACHT4.2, TaTRX-L3.1.1, TaCDSP32.1, and TaNRX1.1 exhibited a thioredoxin fold with central β-sheets surrounded by α-helices, which was in accordance with the conserved structural organization typically reported for the thioredoxin family (Chibani et al., 2018; Le Moigne et al., 2021). TaTRX-L3.1.3 and TaACHT4.2 contained a single thioredoxin domain, with a single redox-active site, WCRKC and GCGGC, respectively (**Figures 5A, B**). This finding was consistent with previous reports showing that plant TRX-like and ACHT proteins belong to the atypical thioredoxin family and are organized as single-domain thioredoxin-like proteins (Chibani et al., 2021). TaNRX1.1 and TaCDSP32.1 contained multiple thioredoxin domains. TaCDSP32.1 contained two thioredoxin domains with a single redox-active site, HCGPC, whereas TaNRX1.1 possessed three thioredoxin domains with two redox-active sites, WCGPC and WCPPC (**Figures 5C, D**). This finding was consistent with previous studies showing that plant CDSP32 proteins are composed of two thioredoxin modules with a single functional active site, whereas plant NRXs are characterized by a multidomain organization containing three thioredoxin-like domains (Rey et al., 2024; Ghafoor et al., 2025).

**Figure 5:**
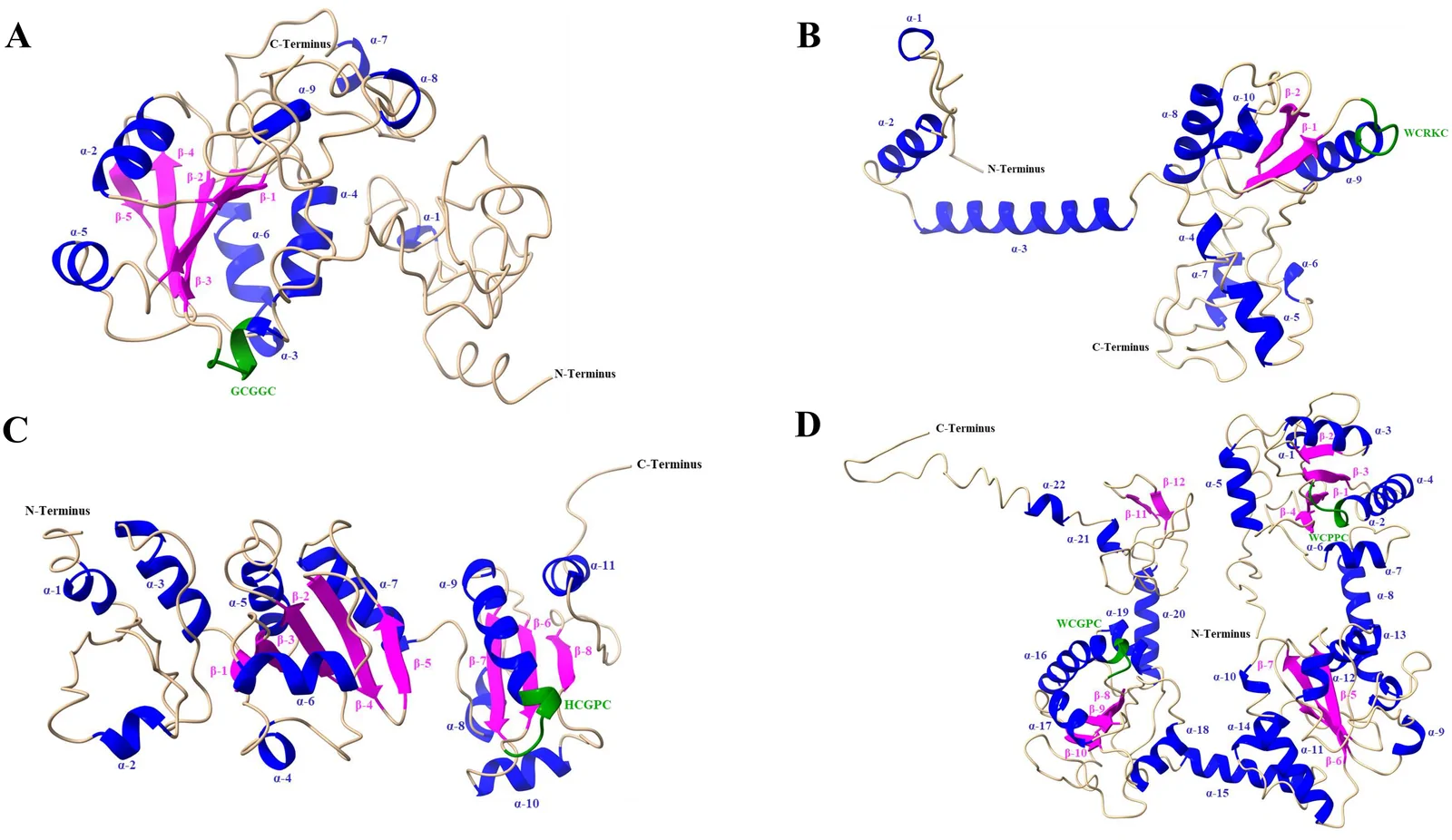
Predicted 3D structures of atypical TRXs. **(A)** TaACHT4.2, **(B)** TaTRX-L3.1.3, **(C)** TaCDSP32.1, and **(D)** TaNRX1.1.

#### 3.4.2 Molecular docking analysis of atypical thioredoxins with hormones

To assess the predicted affinity of the typical and atypical thioredoxin proteins toward the selected hormones, molecular docking was performed between the predicted protein structures (**Figure 5**) and the chemical structures of the hormones (ABA, IAA, and MeJA) shown in **Figure 6**. As ET is a gaseous and nonpolar phytohormone, its intracellular distribution remains poorly documented. Moreover, unlike canonical ethylene receptors, TRXs are not known to contain Cu(I)-dependent ethylene-binding sites. Therefore, the molecular docking analysis was limited to ABA, IAA, and MeJA.

**Figure 6:**
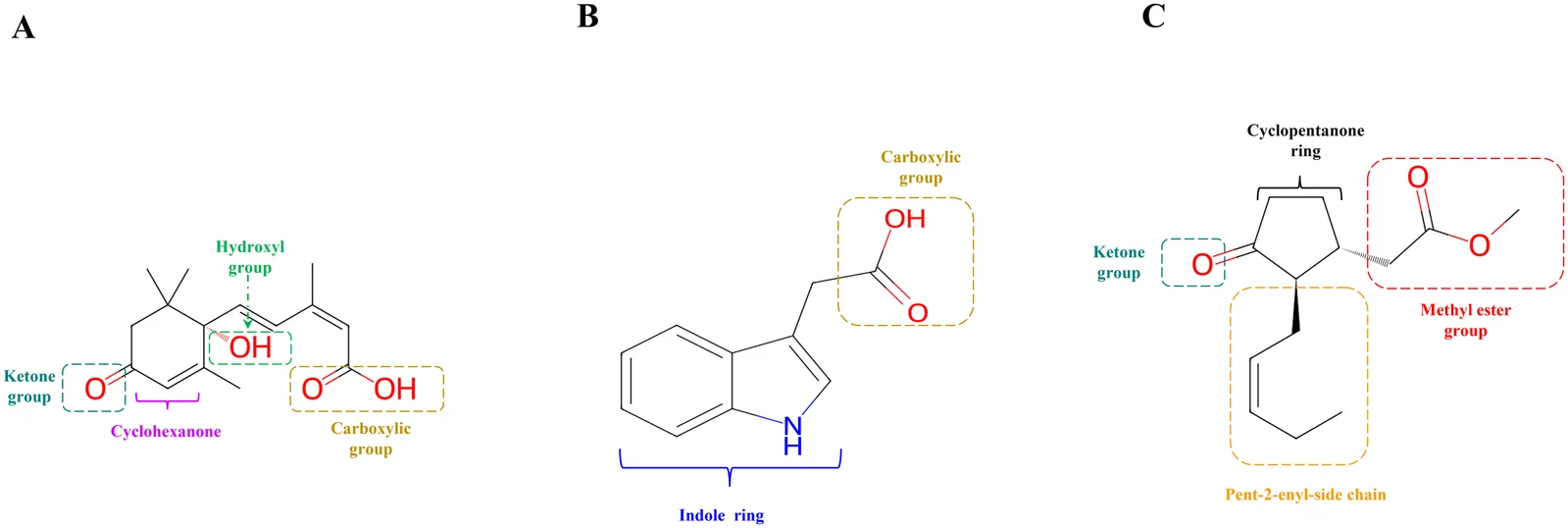
Hormone chemical structures and major functional groups: **(A)** abscisic acid (ABA), **(B)** indole-3-acetic acid (IAA), and **(C)** methyl jasmonate (MeJA).

A molecular docking approach was used to support the hypothesis that the hormone-mediated regulation of thioredoxins may involve direct molecular interactions. Furthermore, Anabuki et al. (2021) identified the typical AtTRX-h3 as an ABA-binding protein using an affinity-based chemical labeling strategy; however, the specific binding sites and affinity were not determined. Molecular docking analysis highlighted the high affinity of TaACHT4.2, TaCDSP32.1, TaTRX-L3.1.3, and TaNTRX1 for ABA and IAA (**Table 3**). TaCDSP32.1 and TaNRX1.1 showed the highest affinity for ABA and IAA. TaCDSP32.1 exhibited binding energies of − 7.5 kcal/mol for ABA and − 7.3 kcal/mol for IAA, whereas TaNRX1.1 showed binding energies of − 7.1 kcal/mol for ABA and − 6.1 kcal/mol for IAA (**Table 3**). The predicted high affinities of TaCDSP32.1 and TaNRX1.1 can be explained by their multidomain organization and protein folds (**Figures 5C, D**). TaCDSP32.1 is a protein with two TRX modules, whereas TaNRX1.1 contains three TRX modules, which provide several pockets to stabilize the large ABA molecule, measuring approximately 11–13 Å, the IAA molecule, measuring approximately 8–9Å, and the MeJA molecule, measuring approximately 10–11 Å.

**Table 3:** Predicted molecular docking affinities of atypical TaTRXs with hormones.

TRX group	Protein name	ABA (kcal/mol)	IAA (kcal/mol)	MeJA (kcal/mol)
Atypical TRX	TaACHT4.2	− 6.6	− 5.9	− 5.5
	TaCDSP32.1	− 7.5	− 7.3	− 6.3
	TaTRX-L3.1.3	− 6.7	− 5.8	− 5.3
	TaNRX1.1	− 7.1	− 6.1	− 6

ACHT, atypical Cys/His-rich thioredoxin; ABA, abscisic acid; CDSP32, chloroplast drought-induced stress protein of 32 kDa; ET, ethylene; IAA, indole acetic acid; MeJA, methyl jasmonate; NRX, nucleoredoxin.

As the molecular docking affinities showed significant results, a binding site analysis was performed to identify the predicted binding sites for ABA, IAA, and MeJA in TaACHT4.2, TaCDSP32.1, TaTRX-L3.1.3, and TaNRX1.1 (**Table 4**). TaACHTA4.2 showed the same binding pocket for ABA and MeJA, whereas IAA was predicted to bind to a different pocket (**Figure 7**). TaCDSP32.1, TaTRX-L3.1.3, and TaNRX1.1 were predicted to bind ABA, IAA, and MeJA within the same regions (**Figures 8**–**10**).

**Table 4:** Predicted molecular binding sites of atypical TaTRXs and hormones.

TRX group	Protein name	Hormones (ligand)	Predicted binding sites
Atypical TRX	TaACHT4.2	ABA	Pro40–Ala41, Met77–Asn78–Leu79, Trp87, Lys90–Gly91–Gln92, His133–Pro134, Cys137–Gln138, Glu141
		IAA	Leu92, Glu98, Arg263–Gln264, Arg269
		MeJA	Pro40–Ala41, Leu79, Lys90–Gly91, Gln93, His133–Pro134, Cys137–Gln138, Glu141
	TaCDSP32.1	ABA	Leu88–Gln89, Lys90–Ala91–Lys92–Asn93, Val96, Val98, Lys141, Glu145, Phe155–Tyr156, Gly158–Thr159, Lys161
		IAA	Val78, Phe84–Leu88–Gln89–Ly90–Ala91–Ly92, Val96–Val97–Val98, Phe100, Lys128, Lys141, Glu145, Ser154–Phe155–Tyr156, Lys161
		MeJA	Leu88–Gln89–Lys90–Ala91–Lys92–Asn93
	TaTRX-L3.1.3	ABA	Ser125–Val126–Pro127–Val128, Leu172–Glu173, Ala176–Glu177, Pro180–Gly181–Val182–Arg183–Phe184, Phe186
		IAA	Ser125–Val126, Val128, Leu172–Glu173, Ala176–Gly177–Glu178–Phe179–Pro180–Gly181–Val182, Phe184
		MeJA	Val126, Val128, Met130, Lys169–Pro170, Leu172–Glu173, Ala176–Gly177, Val182, Phe184, Phe186
	TaNRX1.1	ABA	Thr48, Phe82, Phe133, Asp135, Lys137–Thr138, Leu142–Thr143, Glu145, Phe149, Tyr158–Pro159–Phe160–Thr161, Ile165
		IAA	Thr48, Phe82, Phe133–Leu134–Asp135, Thr138, Leu142–Thr143, Glu145, Tyr158, Phe160, Ile165
		MeJA	Thr48, Lys80, Phe82, Phe133, Asp135, Leu142–Thr143, Phe149–Val150, Tyr158–Pro159–Phe160–Thr161, Ile165

*ACHT*, atypical Cys/His-rich thioredoxin; *ABA*, abscisic acid; *CDSP32*, chloroplast drought-induced stress protein of 32 kDa; *ET*, ethylene; *IAA*, indole acetic acid; *MeJA*, methyl jasmonate; *NRX*, nucleoredoxin.

**Figure 7:**
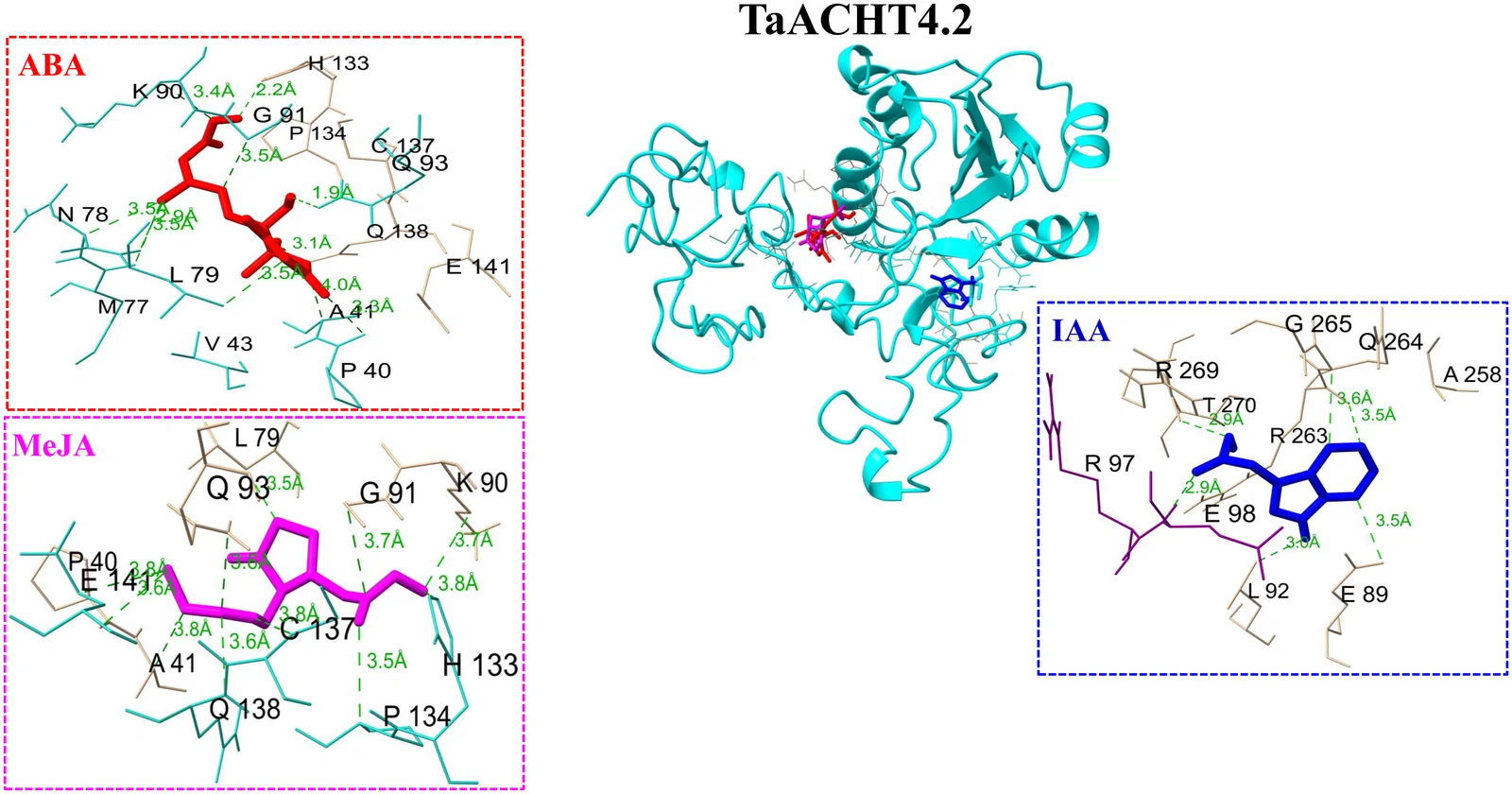
Predicted 3D binding interactions of TaACHT4.2 with abscisic acid (ABA), indole-3-acetic acid (IAA), and methyl jasmonate (MeJA). 3D views depict the amino acid residues involved in hormone stabilization and the predicted interaction obtained by molecular docking.

**Figure 8:**
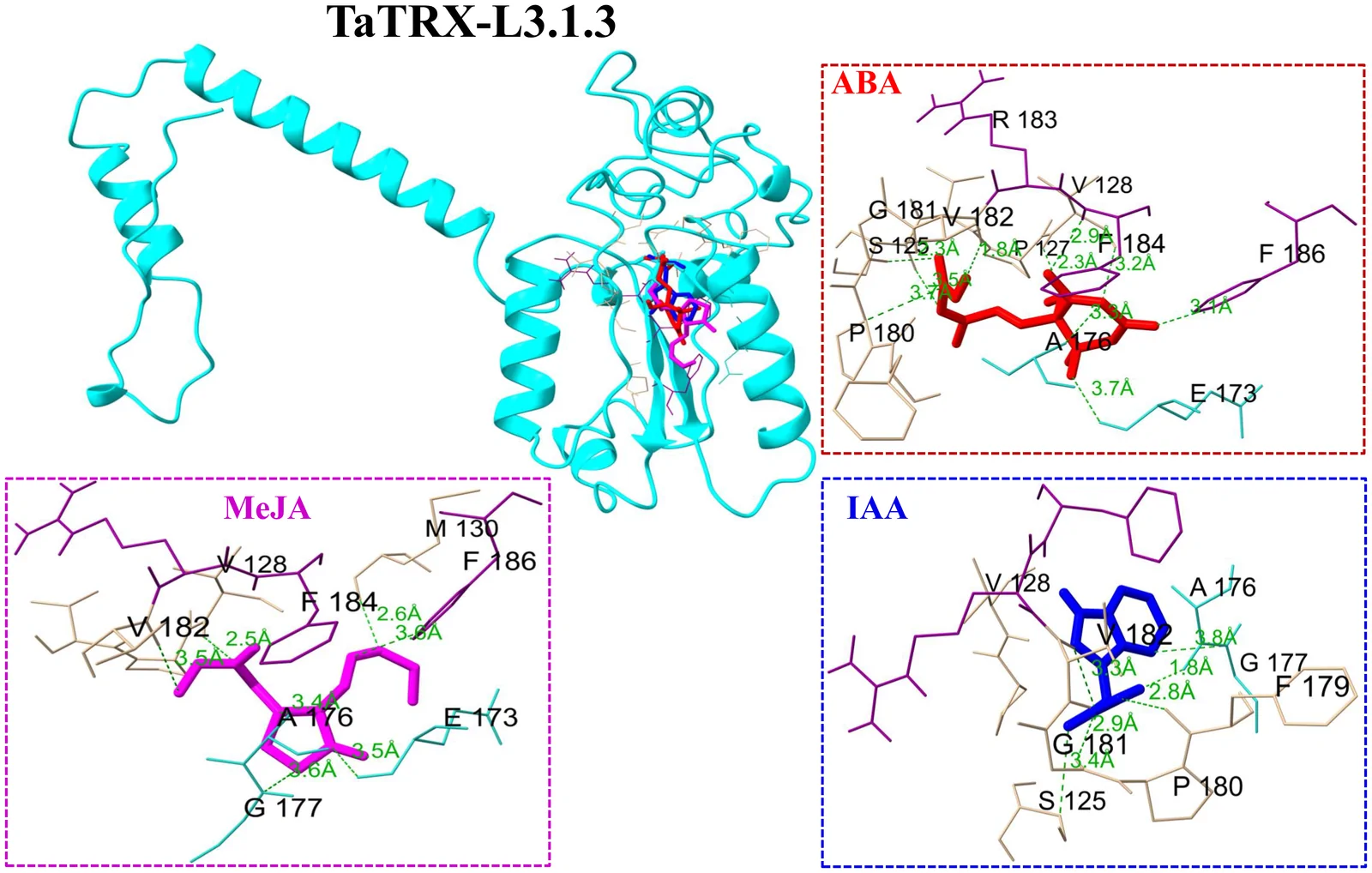
Predicted 3D binding interactions of TaTRX-L3.1.3 with abscisic acid (ABA), indole-3-acetic acid (IAA), and methyl jasmonate (MeJA). 3D views depict the amino acid residues involved in hormone stabilization and the predicted interaction obtained by molecular docking.

**Figure 9:**
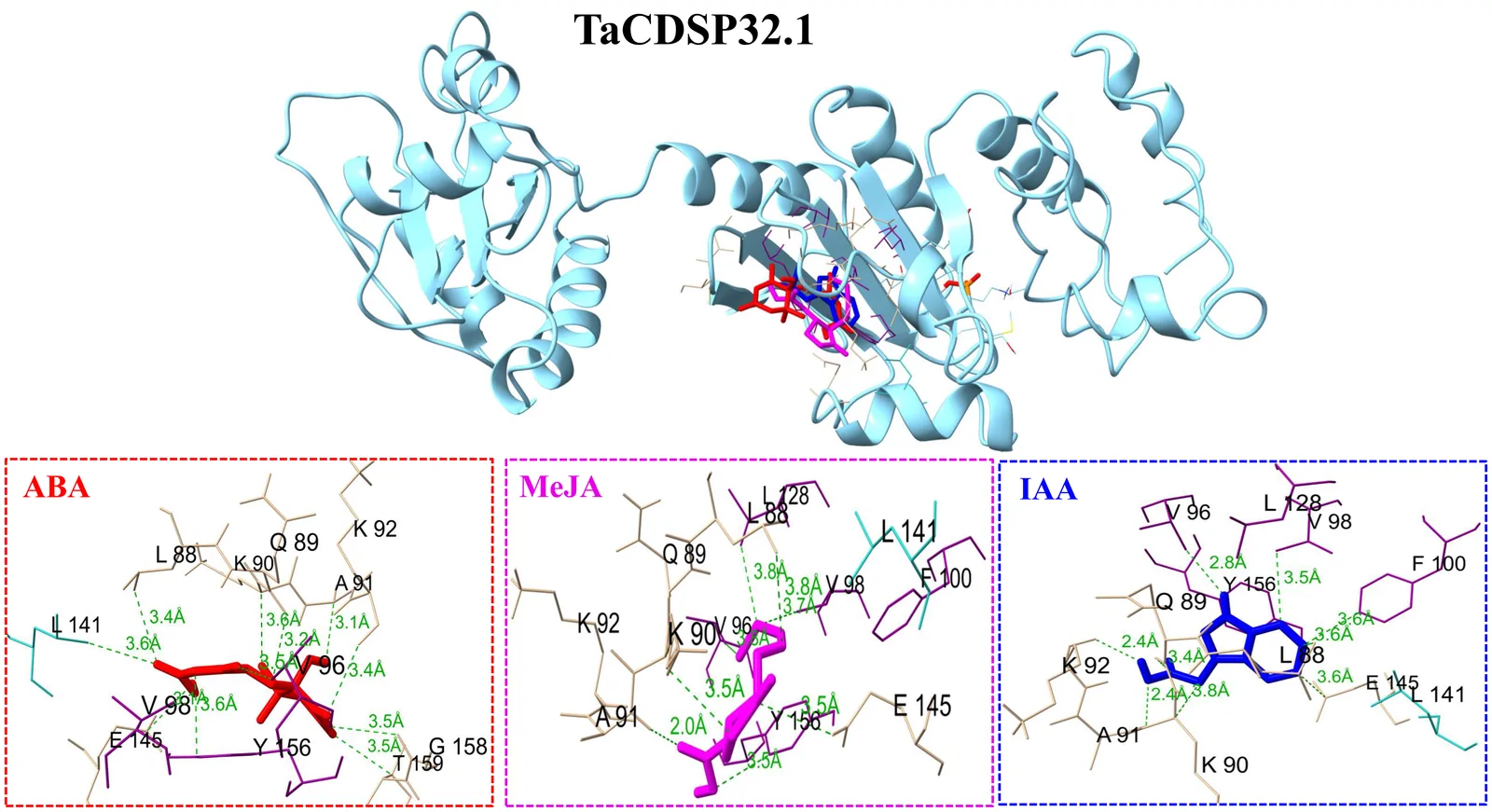
Predicted 3D binding interactions of TaCDSP32.1 with abscisic acid (ABA), indole-3-acetic acid (IAA), and methyl jasmonate (MeJA). 3D views depict the amino acid residues involved in hormone stabilization and the predicted interaction obtained by molecular docking.

**Figure 10:**
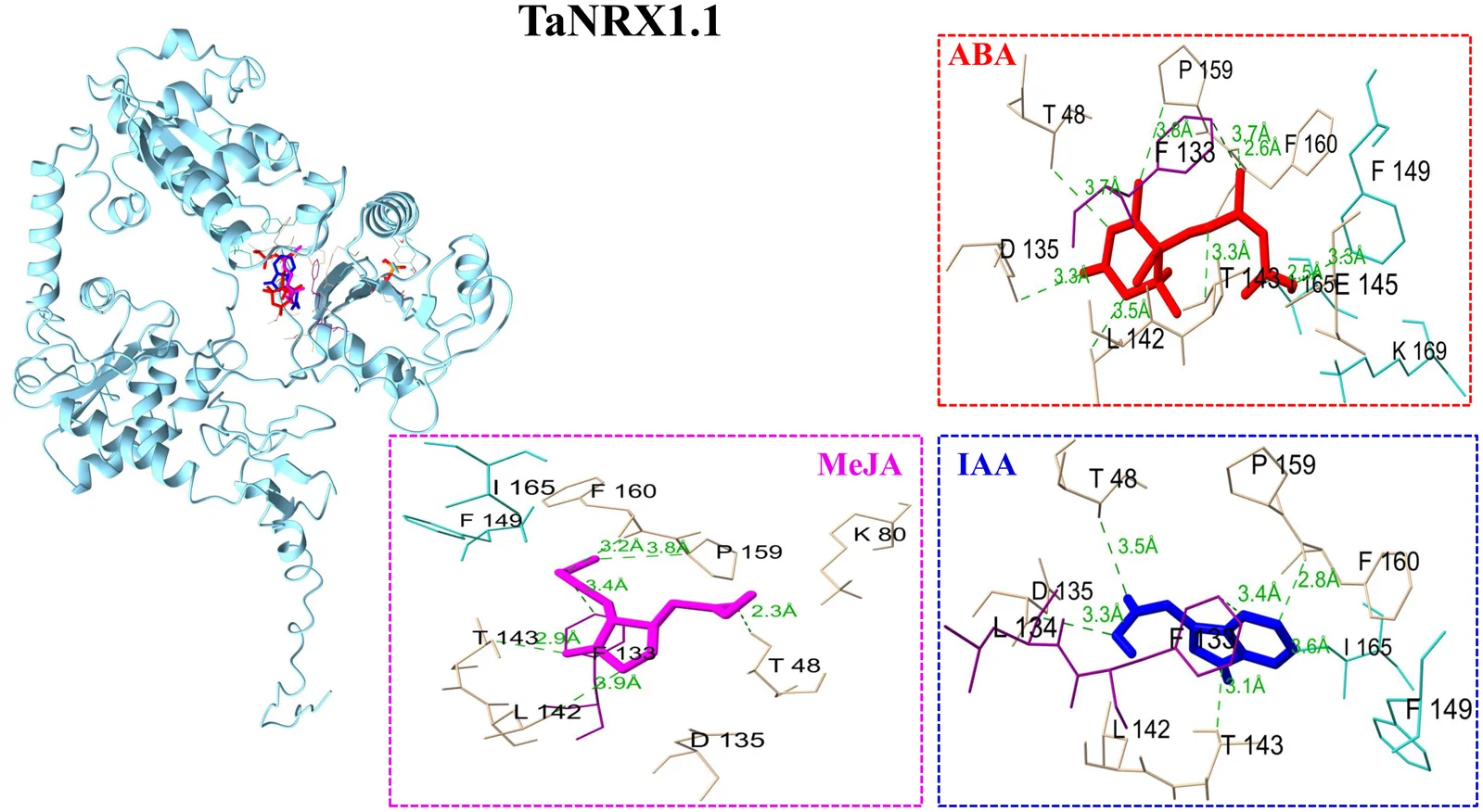
Predicted 3D binding interactions of TaNRX1.1 with abscisic acid (ABA), indole-3-acetic acid (IAA), and methyl jasmonate (MeJA). 3D views depict the amino acid residues involved in hormone stabilization and the predicted interaction obtained by molecular docking.

##### 3.4.2.1 Predicted abscisic acid binding site

ABA is a relatively large isoprenoid phytohormone containing a hydroxyl group, a ketone group, and a carboxylic acid group (**Figure 6A**), which requires a sufficiently diverse binding pocket for stabilization (Liu et al., 2022). The predicted binding site analysis revealed that ABA interacts with the atypical TRXs through pockets composed mainly of neutral, polar, aromatic, and basic amino acids (**Table 4**; **Figures 7**–**10**). TaACHT4.2 contained a mixed polar and hydrophobic pocket, whereas TaCDSP32 was enriched in Lys residues that may stabilize the ABA carboxyl group (**Table 4**). The TaTRX-L3.1.3 and TaNRX1.1 pockets were dominated by hydrophobic and aromatic residues, which may stabilize the nonpolar region of ABA. Among the analyzed proteins, TaCDSP32.1 exhibited the most favorable predicted binding pocket, consistent with its strongest ABA binding affinity of − 7.3 kcal/mol (**Table 3**). Similar interactions involving hydrophobic residues, hydrogen bonds, and conserved Lys residues have been reported in PYR/PYL/PCAR ABA receptors (Liu et al., 2023; Lozano-Juste et al., 2023).

##### 3.4.2.2 Predicted indole-3-acetic acid binding site

IAA is the most abundant natural auxin synthesized by plants and is metabolized and synthesized in several compartments, including plastids, chloroplasts, the cytosol, and the nucleus, while vacuoles act as an important storage compartment for auxin-related metabolites (Friml, 2022; Casanova-Sáez et al., 2026). Nitrilase 1 (NIT1), which catalyzes the conversion of indole-3-acetonitrile (IAN) into IAA, has also been reported to localize to chloroplasts (Ma and Kriechbaumer, 2026). IAA is an aromatic molecule containing a hydrophobic indole ring and a carboxyl group (**Figure 6B**). The predicted binding pockets of TaACHT4.2, TaCDSP32.1, TRX-L3.1.3, and TaNRX1.1 were mainly enriched in hydrophobic and aromatic residues that may stabilize the indole ring, together with polar or charged residues that may interact with the carboxyl group (**Table 4**; **Figures 7**–**10**). TaCDSP32.1 and TaNRX1.1 showed the most extensive hydrophobic and aromatic pockets, whereas Lys and Arg residues in TaACHT4.2 and TaCDSP32.1 may provide additional electrostatic stabilization. These predicted interactions were consistent with known auxin-binding mechanisms, in which IAA is stabilized within hydrophobic pockets through its aromatic indole ring, while polar and charged residues contribute to the binding and stabilization of the carboxyl group (Prieto-Martínez et al., 2024; Yang et al., 2025).

##### 3.4.2.3 Predicted methyl jasmonate binding site

MeJA is a volatile methyl ester derivative of jasmonic acid (JA) that can be converted back to JA by methyl jasmonate esterases (Liu and Timko, 2021). Jasmonate biosynthesis occurs in the chloroplast, whereas JA signaling occurs in the nucleus through the CORONATINE INSENSITIVE 1-JASMONATE ZIM-domain (COI1-JAZ) co-receptor complex (Zhou et al., 2025). MeJA is a predominantly hydrophobic molecule containing a cyclopentanone ring, a pentenyl side chain, and a methyl ester group (**Figure 6C**). The predicted binding pockets of TaACHT4.2, TaCDSP32.1, TaTRX-L3.1.3, and TaNRX1.1 were mainly enriched in hydrophobic and aromatic residues, including Leu, Val, Pro, Ala, Phe, and Tyr, which may stabilize the cyclopentanone ring and pentenyl side chain through hydrophobic, aromatic, and van der Waals interactions (**Table 4**; **Figures 7**–**10**). Polar or charged residues, including Gln, Lys, His, and Glu, may additionally interact with the oxygen-containing groups of MeJA. TaTRX-L3.1.3 and TaNRX1.1 showed the most extensive hydrophobic and aromatic pockets, whereas TaACHT4.2 and TaCDSP32.1 contained smaller and less hydrophobic binding sites. These predicted interactions support the hypothesis that chloroplastic TaTRX-L3.1.3 and nuclear TaNRX1.1 may represent potential MeJA-interacting proteins.

### 3.5 Conclusion and future prospects

This study provides an integrated characterization of typical and atypical thioredoxins and thioredoxin reductases in *T. aestivum*, including their genome-wide identification, classification, hormone-responsive expression patterns, and predicted interactions with phytohormones. Comparative genomic analysis showed that all the classes identified in *A. thaliana* were conserved in the *T. aestivum* genome, although several classes were expanded as a consequence of wheat polyploidy. All the redox centers identified in the protein sequences were fully conserved, except for the TaACHT6-specific SCGSC signature. Hormonal expression profiling revealed that the atypical *TaTRXs* were more responsive than the typical *TaTRXs* and *TaTRs*. *TaACHT4.2*, *TaNRX1.1*, *TaCDSP32.1*, and *TaTRX-L3.1.3* were identified as hormone-responsive candidates. Molecular docking further supported their potential interactions with ABA, IAA, and MeJA, suggesting that these proteins may contribute to hormone-dependent redox processes in chloroplastic and nuclear compartments. These findings establish a genomic and functional basis for understanding the relationship between thioredoxin-mediated redox regulation and phytohormone signaling in wheat and identify promising targets for future biochemical validation and genetic manipulation aimed at improving crop growth, development, and stress resistance.

## Data Availability

The datasets generated or analyzed during the current study are available in the Figshare repository (https://doi.org/10.6084/m9.figshare.32687874). The data supporting this study are publicly available from NCBI GEO: GSE103430. Further inquiries can be directed to the corresponding author/s.

## References

[B1] Anabuki T. Ohashi K. Takasuka T. E. Matsuura H. Takahashi K. (2021). AtTrxh3, a thioredoxin, is identified as an abscisic acid binding protein in *Arabidopsis thaliana*. Molecules 28, 161. doi: 10.3390/molecules27010161 35011393 PMC8746859

[B2] Ancín M. Fernandez-Irigoyen J. Santamaria E. Larraya L. Fernández-San Millán A. Veramendi J. . (2022). New *in vivo* approach to broaden the thioredoxin family interactome in chloroplasts. Antioxidants 11, 1979. doi: 10.3390/antiox11101979 36290702 PMC9598788

[B3] Casanova-Sáez R. Pěnčík A. Brunoni F. Ament A. Hladík P. Žukauskaitė A. . (2026). Comprehensive characterisation of IAA inactivation pathways reveals the impact of glycosylation on auxin metabolism and plant development in Arabidopsis. Commun. Biol. 9, 762. doi: 10.1038/s42003-026-10431-5 42243496 PMC13237164

[B4] Cha J. Y. Ahn G. Jeong S. Y. Shin G.-I. Ali I. Ji M. G. . (2022). Nucleoredoxin 1 positively regulates heat stress tolerance by enhancing the transcription of antioxidants and heat-shock proteins in tomato. Biochem. Biophys. Res. Commun. 635, 12–18. doi: 10.1016/j.bbrc.2022.10.033 36252332

[B5] Chibani K. Pucker B. Dietz K. J. Cavanagh A. (2021). Genome‐wide analysis and transcriptional regulation of the typical and atypical thioredoxins in *Arabidopsis thaliana*. FEBS Lett. 595, 2715–2730. doi: 10.1002/1873-3468.14197 34561866

[B6] Chibani K. Saul F. Didierjean C. Rouhier N. Haouz A. (2018). Structural snapshots along the reaction mechanism of the atypical poplar thioredoxin-like2.1. FEBS Lett. 592, 1030–1041. doi: 10.1002/1873-3468.13009 29453875

[B7] Chibani K. Wingsle G. Jacquot J. P. Gelhaye E. Rouhier N. (2009). Comparative genomic study of the thioredoxin family in photosynthetic organisms with emphasis on *Populus trichocarpa*. Mol. Plant 2, 308–322. doi: 10.1093/mp/ssn076 19825616

[B8] Considine M. Foyer C. (2021). Oxygen and reactive oxygen species-dependent regulation of plant growth and development. Plant Physiol. 86, 79–92. doi: 10.1093/plphys/kiaa077 33793863 PMC8154071

[B9] Dallakyan S. Olson A. J. (2015). Small-molecule library screening by docking with PyRx. Methods Mol. Biol. 1263, 243–250. doi: 10.1007/978-1-4939-2269-7_19 25618350

[B10] Dangoor I. Peled-Zehavi H. Levitan A. Pasand O. Danon A. (2009). A small family of chloroplast atypical thioredoxins. Plant Physiol. 149, 1240–1250. doi: 10.1104/pp.108.128314 19109414 PMC2649386

[B11] De Brasi-Velasco S. Aroca A. Romero L. C. Gotor C. Sevilla F. Jiménez A. (2025). New role for thioredoxins in plants: implication of TRXo1 in protein depersulfidation. Redox Biol. 82, 103627. doi: 10.1016/j.redox.2025.103627 40220625 PMC12018006

[B12] Delorme-Hinoux V. Bangash S. A. Meyer A. J. Reichheld J. P. (2016). Nuclear thiol redox systems in plants. Plant Sci. 243, 84–95. doi: 10.1016/j.plantsci.2015.12.002 26795153

[B13] Emanuelsson O. Nielsen H. Brunak S. von Heijne G. (2000). Predicting subcellular localization of proteins based on their N-terminal amino acid sequence. J. Mol. Biol. 300, 1005–1016. doi: 10.1006/jmbi.2000.3903 10891285

[B14] Friml J. (2022). Fourteen stations of auxin. Cold Spring Harb. Perspect. Biol. 14, a039859. doi: 10.1101/cshperspect.a039859 34400554 PMC9159264

[B15] Fuchs H. Staszak A. M. Vargas P. A. Sahrawy M. Serrato A. J. Dyderski M. K. . (2024). Redox dynamics in seeds of *Acer* spp: unraveling adaptation strategies of different seed categories. Front. Plant Sci. 15, 1430695. doi: 10.3389/fpls.2024.1430695 39114470 PMC11303208

[B16] Geigenberger P. Thormählen I. Daloso D. M. Fernie A. R. (2017). The unprecedented versatility of the plant thioredoxin system. Trends Plant Sci. 22, 249–262. doi: 10.1016/j.tplants.2016.12.008 28139457

[B17] Ghafoor M. S. Naz R. Hussain A. Nosheen A. Yasmin H. Sajjad M. . (2025). Nucleoredoxin 1 in wheat: genomic analysis and demonstration of its role in redox homeostasis and stress resilience. Plant Direct 9, e70130. doi: 10.1002/pld3.70130 41377839 PMC12688404

[B18] Goodstein D. M. Shu S. Howson R. Neupane R. Hayes R. D. Fazo J. . (2012). Phytozome: a comparative platform for green plant genomics. Nucleic Acids Res. 40, D1178–D1186. doi: 10.1093/nar/gkr944 22110026 PMC3245001

[B19] Hägglund P. Finnie C. Yano H. Shahpiri A. Buchanan B. B. Henriksen A. . (2016). Seed thioredoxin h. Biochim. Biophys. Acta 1864, 974–982. doi: 10.1016/j.bbapap.2016.02.014 26876537

[B20] Hastings J. Owen G. Dekker A. Ennis M. Kale N. Muthukrishnan V. . (2015). ChEBI in 2016: improved services and an expanding collection of metabolites. Nucleic Acids Res. 44, D1214–D1219. doi: 10.1093/nar/gkv1031 26467479 PMC4702775

[B21] Horton P. Park K. J. Obayashi T. Fujita N. Harada H. Adams-Collier C. J. . (2007). WoLF PSORT: protein localization predictor. Nucleic Acids Res. 35, W585–W587. doi: 10.1093/nar/gkm259 17517783 PMC1933216

[B22] Iqbal N. Khan N. A. Ferrante A. Trivellini A. Francini A. Khan M. I. R. (2017). Ethylene role in plant growth, development and senescence: interaction with other phytohormones. Front. Plant Sci. 8, 475. doi: 10.3389/fpls.2017.00475 28421102 PMC5378820

[B23] Khan N. (2025). Decoding phytohormone signaling in plant stress physiology: insights, challenges, and future directions. Environ. Exp. Bot. 231, 106099. doi: 10.1016/j.envexpbot.2025.10609942574925

[B24] Kim S. (2021). Exploring chemical information in PubChem. Curr. Protoc. 1, e217. doi: 10.1002/cpz1.217 34370395 PMC8363119

[B25] Kim S. Chen J. Cheng T. Gindulyte A. He J. He S. . (2021). PubChem in 2021: new data content and improved web interfaces. Nucleic Acids Res. 49, D1388–D1395. doi: 10.1093/nar/gkaa971 33151290 PMC7778930

[B26] Kumar S. Stecher G. Suleski M. Sanderford M. Sharma S. Tamura K. (2024). Molecular evolutionary genetics analysis version 12 for adaptive and green computing. Mol. Biol. Evol. 41, 1–9. doi: 10.1093/molbev/msae263 39708372 PMC11683415

[B27] Le Moigne T. Gurrieri L. Crozet P. Marchand C. H. Zaffagnini M. Sparla F. . (2021). Crystal structure of chloroplastic thioredoxin z defines a type-specific target recognition. Plant J. 107, 434–447. doi: 10.1111/tpj.15300 33930214

[B28] Li Y. Xue H. Lu Y. Xiang Z. Hou Z. Zhou Y. . (2025). Genome-wide thioredoxin system in *Cardamine hupingshanensis*: role in Se stress and metabolism. Biology 14, 1404. doi: 10.3390/biology14101404 41154807 PMC12561512

[B29] Liu Y. Chen S. Wei P. Guo S. Wu J. (2022). A briefly overview of the research progress for the abscisic acid analogues. Front. Chem. 10, 967404. doi: 10.3389/fchem.2022.967404 35936098 PMC9355028

[B30] Liu R. Liang G. Gong J. Wang J. Zhang Y. Hao Z. . (2023). A potential ABA analog to increase drought tolerance in *Arabidopsis thaliana*. Int. J. Mol. Sci. 24, 8783. doi: 10.3390/ijms24108783 37240123 PMC10218568

[B31] Liu H. Timko M. P. (2021). Jasmonic acid signaling and molecular crosstalk with other phytohormones. Int. J. Mol. Sci. 22, 2914. doi: 10.3390/ijms22062914 33805647 PMC8000993

[B32] Livak J. Schmittgen T. D. (2001). Analysis of relative gene expression data using real-time quantitative PCR and the 2^-ΔΔCt^ method. Methods 25, 402–408. doi: 10.1006/meth.2001.1262 11846609

[B33] Lozano-Juste J. Infantes L. Garcia-Maquilon I. Ruiz-Partida R. Merilo E. Benavente J. L. . (2023). Structure-guided engineering of a receptor-agonist pair for inducible activation of the ABA adaptive response to drought. Sci. Adv. 9, eade9948. doi: 10.1126/sciadv.ade9948 36897942 PMC10005185

[B34] Mo W. Zheng X. Shi Q. Zhao X. Chen X. Yang Z. . (2024). Unveiling the crucial roles of abscisic acid in plant physiology: implications for enhancing stress tolerance and productivity. Front. Plant Sci. 15, 1437184. doi: 10.3389/fpls.2024.1437184 39640997 PMC11617201

[B35] Nakai K. Horton P. (1999). PSORT: a program for detecting sorting signals in proteins and predicting their subcellular localization. Trends Biochem. Sci. 24, 34–35. doi: 10.1016/s0968-0004(98)01336-x 10087920

[B36] Nascimento C. P. da Fonseca-Pereira P. Ferreira-Silva M. Rosado-Souza L. Linka N. Fernie A. R. . (2025). Functional analysis of the extraplastidial TRX system in germination and early stages of development of *Arabidopsis thaliana*. Plant Sci. 350, 112310. doi: 10.1016/j.plantsci.2024.112310 39477093

[B37] Noctor G. Reichheld J.-P. Foyer C. H. (2018). ROS-related redox regulation and signaling in plants. Semin. Cell. Dev. Biol. 80, 3–12. doi: 10.1016/j.semcdb.2017.07.013 28733165

[B38] Pandey E. Kumari R. Faizan S. Pandey S. (2025). Linking the interaction of salicylates and jasmonates for stress resilience in plants. Stress Biol. 28, 64. doi: 10.1007/s44154-025-00250-9 41148432 PMC12569331

[B39] Panozzo A. Bolla P. Barion G. Botton A. Vamerali T. (2025). Phytohormonal regulation of abiotic stress tolerance, leaf senescence and yield response in field crops: a comprehensive review. Biotech. 14 (1), 4. doi: 10.3390/biotech14010014 40227279 PMC11939854

[B40] Powell H. R. Islam S. A. David A. Sternberg M. J. E. (2025). Phyre2.2: a community resource for template-based protein structure prediction. J. Mol. Biol. 437 (15), 168960. doi: 10.1016/j.jmb.2025.168960 40133783 PMC7617537

[B41] Prieto-Martínez F. D. Mendoza-Cañas J. Martínez-Mayorga K. (2024). To bind or not to bind? A comprehensive characterization of TIR1 and auxins using consensus in silico approaches. Computation 12, 94. doi: 10.3390/computation1205009430654563

[B42] Qi P. F. Jiang Y. F. Guo Z. R. Chen Q. Ouellet T. Zong L. J. . (2019). Transcriptional reference map of hormone responses in wheat spikes. BMC Genomics 20 (1), 390. doi: 10.1186/s12864-019-5726-x 31109305 PMC6528200

[B43] R Core Team (2023). R: A Language and Environment for Statistical Computing (Vienna, Austria: R Foundation for Statistical Computing). Available online at: https://www.R-project.org/ (Accessed February 08, 2026).

[B44] Rey P. Henri P. Alric J. Blanchard L. Viola S. (2024). Participation of the stress-responsive CDSP32 thioredoxin in the modulation of chloroplast ATP-synthase activity in Solanum tuberosum. Plant Cell Environ. 47, 5372–5390. doi: 10.1111/pce.15101 39189948

[B45] Rouhier N. Cerveau D. Couturier J. Reichheld J. P. Rey P. (2015). Involvement of thiol-based mechanisms in plant development. Biochim. Biophys. Acta 1850, 1479–1496. doi: 10.1016/j.bbagen.2015.01.023 25676896

[B46] Salvi P. Manna M. Kaur H. Thakur T. Gandass N. Bhatt D. . (2021). Phytohormone signaling and crosstalk in regulating drought stress response in plants. Plant Cell Rep. 40, 1305–1329. doi: 10.1007/s00299-021-02683-8 33751168

[B47] Sevilla F. Martí M. C. De Brasi-Velasco S. Jiménez A. (2023). Redox regulation, thioredoxins, and glutaredoxins in retrograde signalling and gene transcription. J. Exp. Bot. 74, 5955–5969. doi: 10.1093/jxb/erad270 37453076 PMC10575703

[B48] Souza P. V. L. (2025). The redox power of thioredoxins: evolution, mechanisms, and redox regulation of carbon–nitrogen metabolism in plants. Discover Plants 2, 94. doi: 10.1007/s44372-025-00181-930311153

[B49] Souza P. V. L. Vieira-Neto A. E. (2025). Redefining redox regulation in plants: The evolving role of thioredoxins and TRX-Like 2.2 in plant metabolism. J. Evolutionary Biochem. Physiol. 61, 298–312. doi: 10.1134/s0022093024600362

[B50] Vanneste S. Pei Y. Friml J. (2025). Mechanisms of auxin action in plant growth and development. Nat. Rev. Mol. Cell Biol. 26, 648–666. doi: 10.1038/s41580-025-00851-2 40389696

[B51] Wang X. Wen H. Suprun A. Zhu H. (2025). Ethylene signaling in regulating plant growth, development, and stress responses. Plants (Basel) 21, 309. doi: 10.3390/plants14030309 39942870 PMC11820588

[B52] Yang Z. Wei H. Gan Y. Liu H. Cao Y. An H. . (2025). Structural insights into auxin influx mediated by the Arabidopsis AUX1. Cell. 24, 3960–3973.e15. doi: 10.1016/j.cell.2025.04.028 40378849

[B53] Yu Z. Zhang F. Friml J. Ding Z. (2022). Auxin signaling: Research advances over the past 30 years. J. Integr. Plant Biol. 64, 371–392. doi: 10.1111/jipb.13225 35018726

[B54] Zhang Y. Berman A. Shani E. (2023). Plant hormone transport and localization: Signaling molecules on the move. Annu. Rev. Plant Biol. 22, 453–479. doi: 10.1146/annurev-arplant-070722-015329 36889002

[B55] Zhang J. Li X. Han X. Liu R. Fang J. (2017). Targeting the thioredoxin system for cancer therapy. Trends Pharmacol. Sci. 38, 794–808. doi: 10.1016/j.tips.2017.06.001 28648527

[B56] Zhou K. Han T. Pan B. Hu X. Chen X. Liu X. . (2025). Robustness in jasmonate signaling: Mechanisms of concerted regulation and implications for crop improvement. aBIOTECH 29, 618–637. doi: 10.1007/s42994-025-00244-1 41312099 PMC12647438

[B57] Zhou J. Song T. Zhou H. Zhang M. Li N. Xiang J. . (2022). Genome-wide identification, characterization, evolution, and expression pattern analyses of the typical thioredoxin gene family in wheat (*Triticum aestivum* L.). Front. Plant Sci. 22, 1020584. doi: 10.3389/fpls.2022.1020584 36618641 PMC9813791

