# Supplementary material for: Thioredoxins as hormone-responsive redox regulators in wheat: transcriptomic and structural insights
Source: Front Plant Sci. 2026 Oct 8;17:1911123. doi: 10.3389/fpls.2026.1911123 (PMC13687799; doi:10.3389/fpls.2026.1911123)
Supplement: Supplementary file 1 [file DataSheet1.pdf]

Supplementary material 1: Wheat TRX protein sequences.

TRX-F

>TaTRX-f1.1 [PHYTOZOME:Traes_3AL_6420E8378.1]
MALRLSVSSPQSTASSPAISSCRPAACGRFLACVAASQKRSLMAVSASDGRGVAPVKSG
GLETATTGAGEVEAPTAAVAVTGQVTEVCKDTFWPIVKAAGEKPVVLDMYTQWCGPCKV
MAPKFQEMSEKDHDVVFLKLDCNQDNRPLAKELGIRVVPTFKIFKDGKVAKEVTGAKID
ELARAIEEVKSS

>TaTRX-f1.2[PHYTOZOME: Traes_3B_3C949D318.1]
MALRLSVSSPQSTASSPAISSCRPAACGRFLACVAASQKRSLMAVSASDGRGVAPVKSG
GLETATTGAGEVEAPTAAVAVTGQVTEVCKDTFWPIVKAAGEKPVVLDMYTQWCGPCKV
MAPKFQEMSEKDHDVVFLKLDCNQDNRPLAKELGIRVVPTFKIFKDGKVAKEVTGAKID
ELARAIEEVKSS

>TaTRX-f1.3 [PHYTOZOME:Traes_3DL_07178E28A.1]
MALRLSVSSPQSTASSPAISSCRPAACGRFLACVAASQKRSLMAVSASDGRGVAPVKSG
GLETATTGAGEVEAPTAAVAVTGQVTEVCKDTFWPIVKAAGEKPVVLDMYTQWCGPCKV
MAPKFQEMSEKDHDVVFLKLDCNQDNRPLAKELGIRVVPTFKIFKDGKVAKEVTGAKID
ELARAIEEVKSS

>TaTRX-f2 [PHYTOZOME: Traes_3B_3C949D318.1]
MALRLSVSSPQSTASSPAISSCRPAACGRFLACVAASQKRSLMAVSASDGRGVAPVKSG
GLETATTGAGEVEAPTAAVAVTGQVTEVCKDTFWPIVKAAGEKPVVLDMYTQWCGPCKV
MAPKFQEMSEKDHDVVFLKLDCNQDNRPLAKELGIRVVPTFKIFKDGKVAKEVTGAKID
ELARAIEEVKSS

TRX-h

>TaTRX-h1.1 [PHYTOZOME:Traes_1BL_B1C10EA93.1]
MAEEGKVIAIHSQEQWSTKIEEAGSSTNLVVIDFTATWCGPCRFISPILDEIAKKLPNV
MFLKVDVDEMKSIAEEFSVEAMPTFVLMKEGKVVDKVVGAAKEELSGKIAKHSATACAK

>TaTRX-h1.2 [PHYTOZOME:Traes_1DL_DF3CA9CCC.2]
MQIEEANAAKKLVVVDFTASWCGPCRIMAPVFADLAKKFPNAVFLKVDVDELKPIAEQF
SVEAMPTFLFIKEGDVKDRVVGAIKEELTNKVGLHAAAQ

>TaTRX-h1.3 [PHYTOZOME:Traes_1AL_11FF51731.1]
MAAAATATTTAAATAAAVGPGEVISVHSLEQWTMQIEEANAAKKLVVIDFTASWCGPCR
IMAPIFADLAKKFPAAVFLKVDVDELKPIAEQFSVEAMPTFLFMKEGDVKDRVVGAIKE
ELTTKVGLHAAA

>**TaTRX-h1.4** [PHYTOZOME: Traes_1BL_AF4C87032.1]
MAASAATATATAAAVGAGEVISVHSLEQWTMQIEEANAAKKLVVIDFTASWCGPCRIMA
PIFADLAKKFPAAVFLKVDVDELKPIAEQFSVEAMPTFLFMKEGDVKDRVVGAIKEELT
TKVGLHAAQ

>**TaTRX-h1.5** [PHYTOZOME:Traes_2AL_0ADBF3D27.1]
MAAEEGAVIACHTKQEFDTHMANGKETGKLVIIDFTASWCGPCRVIAPVFAEYAKKFPG
AIFLKVDVDELKDVAEAYNVEAMPTFLFIKDGAKVDTVVGGRKDDIHTKIVALMGSASA

> **TaTRX-h1.6**[PHYTOZOME:Traes_2BS_9C3ACD499.1]
MAAAEEGAVIACHTKQEFDTHMANGKETGKLVIIDFTASWCGPCRVIAPVFAEYAKKFP
GAIFLKVDVDELKDVAEAYNVEAMPTFLFIKDGAKVDTVVGGRKDDIHTKIVALMGSAS
A

>TaTRX-h2.1 [PHYTOZOME:Traes_5DL_27BC0CE09.1]
MGSFLSSLWTPPPLAGDDPDSAVVAVHSKPAWDKHWEEHRNASKLMVIDFSASWCGPCR
FIEPAFKEMASRFADALFVKIDVDELAEVAKTFRVEAMPTFVLVKGGQEVSRVVGAKKD
ELDRKIKTFISST

>TaTRX-h2.2 [PHYTOZOME: Traes_5BL_C6D32C625.1]
MGSFLSSLWTPPPLPGDDPDSAVVAVHSKPAWDRHWEAHRNASKLMVIDFSASWCGPCR
FIEPAFKEMASRFADALFVKIDVDELAVPTFSFLVLLLASLLGFRARSTQLLIGLGIRG
IGPLV

>TaTRX-h9 [PHYTOZOME:Traes_1DS_B39E8C867.1]
MGGCVGKGRSIVEEKLDFKGGNVHVITTKEDWDQKIEEANKDGKIVVANFSASWCGPCR
VIAPVYAEMSKTYPQLMFLTIDVDDLMDFSSTWDIRATPTFFFLKNGQQIDKLVGANKP
ELEKKVQALGDGS

TRX-m

>TaTRX-m1.1 [PHYTOZOME:Traes_2BL_1579D2408.1]
MKTVPVVLLVNSWIPDVTKTTWQSLVIESEIPVLVEFWASWCGPCKMIEPVIGKLSKEY
EGKLKCYKLNTDENPDIASQYGVRSIPTMMIFKNGEKKDAVIGAVPESTLITCIEKFTE

>**TaTRX-m1.2** [PHYTOZOME:Traes_2DL_62BDE92C31.2]
[KEGG:123055193]
MASAPSTTASTLAPPLRSPLSFSSSRRLPSAAALPRAAGALLLCRAPARGLALARVRCR
GAVKLVGEGEFEAEVMKTVPVVLLVNSWIPDVTKTTWQSLVIESEIPVLVEFWASWCGP
CKMIEPVIGKLSKEYEGKLKCYKLNTDENPDIASQYGVRSIPTMMIFKNGEKKDAVIGA
VPESTLITCIEKFTER

>TaTRX-m2 [PHYTOZOME:Traes_5BS_B72CD04F2.1]

MALETCLRGWALHAPQAGIRERLSSGSYAPSRPRTAAPAVVSPSPYKYALVAARRPSRF
VCKCKNVVDEVIVADEKNWDNMVIACESPVLVEFWAPWCGPCRMIAPVIDELAKDYVGK
IKCCKVNTDDCPNIASTYGIRSIPTVLMFKDGEKKESVIGAVPKTTLCTIIDKYIGS

TRX-o

>TaTRXo1 [PHYTOZOME:Traes_7AL_5AD2BD98D.1]
MARRLCRLPRLLLPLAAAPRPQPRLPALNPSPAALLRPSSSPTPTPSFPAFPRLFSSSS
AGDSSMVVVGSADSFEDIHAKVQVEKLPAVFYYTAVWCGPCRAMAPVIEKMSRQYPKIP
VYKVDIDMEGLGTRLSNLKIFSVPTFHFYHKGEKSSEVVGADVKKLEAAMESLHKQQ

>TaTRXo2 [PHYTOZOME:Traes_7BL_BF777344B.1]
MARRLCRLPRLLLPLAAAPKPQPRLPALNPSPAAHLRPSPSSPTLTPSLSAFPRLFSSS
SAGDSSMVVVGSADSFEDIHAKVQVEKLPAVFYYTAVWCGPCRMMAPVIEKMSRQYPKI
PVYKVDIDMDGLGTRLSNLKIFSIPTFHFYHKGEKSSEVVGADVKKLEAAMESLHKQQ

TDX

>TaTDX1 [PHYTOZOME:Traes_5DL_CAF80DFD8.2]

MAKAGENSFEDEIMESDIELEGEVVEPDNDPLQKMGDPSVEVSEEMRDKAQLYKKKGVD
ALSEGKLDEAVEHLTEAILLNPTSAILYAARAGVFVKMKKPNAAILDAEAALQINPDSA
KGYKSRGMAKAMLGKWEDAAHDLHLAAKLDFDEEISSELKKVEPNVHKIEEHKKRYERL
RKERDMKKADLERQRRHAEEVSAASAVLKPGDVITIHSSNQLEEIFTAASKLSKLVILY
FTATWCGPCRFMGPVYKSLSEQHRNVIFLKLDIDQRSNIARRWNVSSVPTFSCVINGKE
IDKVVGADKTGLERKIAEHGSRKQ

>TaTDX2 [PHYTOZOME:Traes_5AL_097E0BB13.1]
MAKVGENSFEDEIMESDIELEGEVVEPDNDPLQKMGDPSVEVSEEMRDKAQLYKKKGVD
ALSEGKLDEAVEHLTEAILLNPTSAILYAARAGVFVKMKKPNAAILDAEAALQINPDSA
KGYKSRGMAKAMLGKWEDAAHDLHLAAKLDFDEEISSELKKVEPNVHKIEEHKKKYERL
RKERDMKKADLERQRRHAEETHILFCDVLSGDVITIHSSNQLEEIFTAASKLSKLVILY
FTATWCGPCRFMGPVYKSLSEQHRNVVFLKLDIDQQGNIAHRWNVSSVPTFSCVINGKE
IDKVVGADKTGLERKIAEHGSRKQ

TRX-y

>TaTRX-y1.1 [PHYTOZOME:Traes_3B_4FCE14A4A.3]
MAAFTTTAAAAPAAYAVARSPAPSPSQWAPTRRSPPAVGLRRAAAPPRRGAALLVKAKK
ETFSTFDELLEKSEKPLLVDFYATWCGPCQYMVPILQEVHEKMSDKINIVKIDTEKYTS
IANKYKIEALPTFIIFKDGEPCYRFEGALPADQLILQIESALEAPK

>TaTRX-y1.2 [PHYTOZOME:Traes_3DL_750FC4166.3]
MAAFTTTAAPSAYAAAAVARSPAPSPSQWAPPHRSPPAVGLRREAAPPRRGAALLVKAK
KETFSTFEELLEKSEKPLLVDFYATWCGPCQYMVPILQEVHEKMSDKINIVKIDTEKYT
SIANKYKIEALPTFIIFKDGEPCYRFEGALPADQLILQIESALEAPK

ACHT

>TaACHT2.1 [PHYTOZOME:Traes_1DS_366988061.2]
MAEALLHHLPRRFGASTTASSSPPSHGVLSPAPRADLRVTRLAAAAPPGRPRRLMAHAA
VRSEAGEQPKWWEKNAGANMIDIHSTQEFLDALRDAGDRLVIVEFYGTWCGSCRALFPR
LCRTATDNPNIVFLKVNFDENKPMCKRLNVKVLPFFHFYRGADGQLEAFSCSLAKFQKL
KDAIAVHNTDRCSIGPPVGVGDVLDSSSSQEKPAEAAS

>TaACHT2.2 [PHYTOZOME:Traes_1AS_37383FD3C.1]
MAEALLHHLPRRFGASTTASSSPSSHGVLSPAPRADLRVTRLAAAAPPGRPHRLMAHAA
VRSETGEQPKWWEKNAGANMIDIHSTQEFLDALRDAGDRLVIVEFYGTWCGSCRALFPR
VITINSY

>TaACHT2.3 [PHYTOZOME:Traes_1BS_C19C8A810.2]
MAEALLHHTATISDPRSSPHRNQGGGGDGRSRLVPHTARLQRRLLGCSRLRYQIVRCLR
RRSRCGRRSRWPRPGATQSTWPRPSSTTSPAASALPPPHPRRRPPTWSSHPPRAPTCGS
RAWRLPPRLGGHAGSWAHAAVRSETGEQPKWWEKNAGANMIDIHSTQEFLDALRDAGDR
LVIVEFYGTWCGSCRALFPRVITTNSY

>TaACHT4.1 [PHYTOZOME:Traes_2DS_5A8D0F48E.1]
MASALCGGGGSVAAPCGDLGAAAALAESLPMGAGYRSKSSFPAGRVALADRPLPRSLQA
AAAAGQMNGNLTIGKAMRWWEKVTHPNMREVESAQDLAYSLLNAGDKLVVVDFFSPGCG
GCRALHPKIAQFAERNPDVLFLQVNYEKHKSMCYSLHVHVLPFFRFYRGAQGRVSSFSC
TNATIKKFKDALAKHSPDRCSLGPARGLEEAELLALAANRDLEFTYNEKPTLVPIAEAI
QMEAASIGGPWMPLPAAATQPLTLGSENGSLIPSGR

>TaACHT4.2 [PHYTOZOME:Traes_4AS_B1D4048C4.1]
MATVQAVAKGTLVSPCGNRAAGFGARRRGAVAARMSPCAPAAVRIGRKSPFLGARLTVG
PRRSKLVSRNLVASPVQMNLAFAKSTKWWEKGLQPNMREIESAQDLVDSLANAGDRLVV
VDFFSPGCGGCRALHPKICQFAEQNPDVLFLQVNYEEHKSMCYSLHVHVLPFFRFYRGA
QGRLCSFSCTNATIKKFRDALAKHNPDRCSIGPTRGLEESELLALAANKDLQFTYTKLP
ESVPSGDSEVIAPGSPRLPPPAKPLVRQGSGERTLVSSGR

>TaACHT6.1[PHYTOZOME:Traes_2AL_63BE91CB7.1]
MIGSFLPLPTTTTSGRCLPPRATATTTLPFPTAPLRIRRRRIRVSVSSSSESKEEGAAS
TNGSLPGLPPVGEEEEEDESCPVECVTEFKTDEEFSRFLERSRATGALVVVDFFRPSCG
SCKYIEQGFMKLCKGSGDHGSPVIFLKHNVIDEYDEQSEVADRLRIKVVPLFHFYKAGV
LVESFATRDKDRITAAIAKYTQPDEHFPFAGRS

>TaACHT6.2 [PHYTOZOME:Traes_2DL_FFF62E69D.1]
MIGSFLPLPTTITTTSGRPLPPRATTTTLPFPTTPLRLGRRQIRVSVSSSSSESNEEGV
GSTNGSLPGLPPVGEEEEEDDESCPVECVTEFKTDEEFSRFLERSRATGALVVVDFFRP
SCGSCKYIEQGFMKLCKGSGDHGSPVIFLKHNVIDEYDEQSEVADRLRIKVVPLFHFYK
AGVLVESFATRDKERITAAIAKYTSPEQEPETEPEE

>TaACHT6.3 [PHYTOZOME:Traes_2BL_D82F44AF2.1]
MIGSFLPLPTTSGRSLPPRATTTTTLPFPRSPLRLRRRQIRVSVSSSSSSESNEEGAG
STNGSLPGLAPVNGEEEEEEEDESCPVECVTEFKTDEELGRFLERSRATGALVVVDFFR
PSCGSCKYIEQGFMKLCKGSGDHGSPVIFLKHNVIDEYDEQSEVAERLRIKVVPLFHFY
KAGVLVESFATRDKDRITAAIAKYTSPDEQEPETQPEE

>TaACHT6.4 [PHYTOZOME:Traes_2BL_D82F44AF2.1]
MIGSFLPLPTTSGRSLPPRATTTTTLPFPRSPLRLRRRQIRVSVSSSSSSESNEEGAG
STNGSLPGLAPVNGEEEEEEEDESCPVECVTEFKTDEELGRFLERSRATGALVVVDFFR
PSCGSCKYIEQGFMKLCKGSGDHGSPVIFLKHNVIDEYDEQSEVAERLRIKVVPLFHFY
KAGVLVESFATRDKDRITAAIAKYTSPDEQEPETQPEE

CDSP32

>TaCDSP32.1 [PHYTOZOME:Traes_6DL_C6E63ED1C.1]
MASTAQLLGHLASSTATASLSFTPVSGGGSSSKVRFLPATAQRRRMVARPRAAVSGAEKEKAKP
AESGGKEDERVVQVHSAEEFDSALQKAKNRLVVVEFAASHSVNSKRIYPCMVDLSRTCGDVDFL
LVMGDESEATRELCQREGITQVPHFSFYKGTEKVHEEEGIGPDQLAGDVLYYGDNHAGVVQLHS
RTDVEALMAEHSGEGGKLLVLDVGLKHCGPCVKVYPTVVKLSRSMADTAVFARMNGDENDACMQ
FLKDMEVVEVPTFLFIRDAKIVGRYVGSGKGELIGEILRYQGVRVTY

>TaCDSP32.2 [PHYTOZOME:Traes_6AL_30768AD47.1]
MASTAQLLGHLAGSTTTASLSVTPVGGGSSKVRFLPAKAQRRRVVARPRAAVSGAEKEKAKPAG
KEDERVVQVHSAEEFDSALQKAKNRLVVVEFAASHSVNSKRIYPCMVDLSRTCGDVDFLLVMGD
ESEATRELCKREGITQVPHFSFYKGTEKVHEEEGIGPDQLAGDVLYYGDNHAGVVQLHSRTDVE
ALMAENSGEDGKLLVLDVGLKHCGPCVKVYPTVVKLSRSMADTAVFARMNGDENDACMQFLKDM
EVVEVPTFLFIRDKKIVGRYVGSGKGELIGEILRYQGVRVTY

CLOT

>TaClot [PHYTOZOME:Traes_7DS_CA226F6E9.1]
MTVEKVDATVADFDGHFERLFAGDDGGKLKLLLFLADREPSSSLTWCPDCNVAEPVIYE

KLESLGKDVVLLRAFVGDKPTWRDPAHPWRVDPRFALKGVPTVLRWDKGAAAGRLEDDE
ANVGDKIDALLN

CXXS

>TaCXXS1.1 [Traes_2AL_CADDB1089.1]
MDVEIQRPREVGSCRVVKVDKEAAWELFTTQAANEGRTVAAHFGASWCVTSLSMNYKFE
ELAQTHPDMLFLFVDVDDVPGVSSKLGVKAMPTFFLIKGKEVVKKIVGANPDELHKMVD
ASDDDSLGSAVTTLPDIVIEK

>TaCXXS1.2 [Traes_2DL_768C1252D.1]
MDAEIQRPREVGSCRVVKVDKEAAWDLFTTQAANEGRTVAAHFGASWCVTSLSMNYKFE
ELAQTHPDMLFLFVDVDDVPGVSSKLGVKAMPTFFLIKGKEVVKKIVGANSDELHKMVD
ASDDDSLGSAVTTLPDIVIEK

TRX-C3

>TaTRX-C3 [PHYTOZOME:Traes_3B_2A134A49A.1]
MATMAGRYCCSYSSPSASAPPPHRQRGPPLQPRPTGGRGRGLRRPSLRAVASAAAEPVK
AATDAEFFQPADSRPIMLFDGVCNLCNGGVRFVREHDPGRSIRYIPLQSDSGRKLLRRS
GRSPDDISSVVLVEKDRSYIKSDAVLRIMEYLNLPFPQLAAFLKIAPLFVRDFAYDNVA
NNRYVVFGRSETESCEIL

TRX-L3

>TaTRX-L3.1.1 [PHYTOZOME:Traes_6BL_69E800C48.1]
MPLLAPGPRALCRDLYCAASAREQAAPVPSSVAACSTVGGALWRGGVRVARTGGRGVRA
EAAYFWDASVPVEMASWCRKCIYLKPKLEKIAGEFPGVRFYFVDVNKVPQAVVKRGNIS
FIYLNSVIAPICLCIYLQKMPTIQLWKDGEWKEEVIGGHKAWLVMDEVREMIQKYK

>TaTRX-L3.1.2 [PHYTOZOME:Traes_6DL_3D072DB07.1]
MPLLAPGPRALCRDLYRAAAAREQAVPSSVTACSTVGGARAVGLRRGGVGGGARTGRGG
VRAEAAYFWDASVPVEMGEIDSMDKLDAALASSAEQNQPIIIDWMASWCRKCIYLKPKL
EKIAGEFPGVMFYFVDVNKVPQAVVKRGNISKMPTIQLWKDGEWKEEVIGGHKAWLVMD
EVREMIHKYK

>TaTRX-L3.1.3 [PHYTOZOME:Traes_6AL_768707567.2]

MPLLAPGPRALCRDLYCAASAREQAAPVPSSVAACSTVGGALWRGGVRVARTGGRGVRA
EAAYFWDASVPVEMGEIDSMDKLDAALASSAEQNQPIIIDWMASWCRKCIYLKPKLEKI
AGEFPGVRFYFVDVNKVPQAVVKRGNISFILILSWLPFVSASAFRKCPQFSCGRMENGR
RK

>TaTRX-L3.3.1 [PHYTOZOME:Traes_2BL_335F9A703.1]
MSAEAGNGKEEARKTGLEGTRLPLPGGSHGSVRCAGSDPQLRHMLDSLKSSKASAVINY
GASWCGVCNQILPPFCKFSNEFKNLSFIYAHVDECPETTQNIRYTPTFHFYRDGERVDE
MFGAGEERLRDRLWLHS

NRX

>TaNRX1.1 [PHYTOZOME:Traes_2DL_28DFAC79D.2]
MAASSPTPGGGLGAILAAGDRDYLIRNSGEQVKISSIEGDTVAIYFSASWCPPCQRFTP
KLIEAYKELTSHGKSFEVIFVSGDQDEEAFNAYFAKMPWLAVPFSDSEGRKSLDERFEV
NGIPHLVFLDAKTGEVLTDEGVEFVSEYGIEAYPFTTERINELKEQEKAAKDNQTIHSV
LATPNRNYVISNTGEKVPIVDLEGKYVGICFVVNGYPPVEEFTPVLAKIYAKLKEVGEK
FEVVAVSLDSDEESFNNSFSSMPWLAIPQGDKMCQKLVSYFELSDLPTLVLIGPDGKTL
SSNIADIINEHGLDAWEGFPFNAEKLEILAEKAKAKAASQTLESLLVTGDVDFVIGKDG
AKVPVAELVGKTVLLYFSAKWCGPCRAFLPTLVDVYNKIKEKNSDFEIVFISSDRDQSS
FDDFFSGMPWLAIPLEDERKAYLKKKFKIRGIPSLVAIGPDGKTVNTDAKTSLAVHGAD
AFPFTSERIQELEKKIDEMAKGWPEKVKHELHEHELVLIRRPRPYGCDGCEEMGTSWSY
NCAECDFDLHAKCALGEEKKGEEVNGQEHAAAPAGYVCEGDVCRKA

>TaNRX1.2 [PHYTOZOME:Traes_2AL_D80447132.1]
MAASPTPGGGLGAILAAGDRDYLVRNSGEKQVKISSIEGDTVAIYFSASWCPPCQRFTP
KLIEAYTELTSNGKSFEVIFVSGDRDEEAFNAYFAKMPWLAVPFYDSEGRKSLDERFDV
NGIPHLVFLDAKTGEVLTDEGVEFVSEYGIEAYPFTTERINELKEQEKAAKDNQTIHSV
LATPNRNYVISNTGEKVPIVDLEGKYVGICFVVNGYPPVEEFTPVLAKIYAKLKEVGEK
FEVVAVSLDSDEESFNNSFSSMPWLAIPHGDKMCQKLVSYFELSDLPTLVLIGPDGKTL
SSNIADIINEHGLDAWEGFPFNAEKLEILAEKAKAKAASQTLESLLVTGDVDFVIGKDG
AKVPVAELVGKTVLLYFSAKWCGPCRAFLPTLVDVYNKIKEKNSDFEIVFVSSDRDQSS
FDDFFSGMPWLAIPLEDERKAYLKKKFKIRGIPSLVAIGPDGKTVNTDAKTSLAVHGAD
AFPFTDERIQELEKKIEEMAKGWPEKLKHELHEHELVLIRRPRPYGCDGCEEMGTSWSY
NCAECDFDLHTKCALGEEKKGEEVNGQEHAAAPAGYVCEGDVCRKA

>TaNRX1.3 [PHYTOZOME:Traes_2BL_34819D129.1]
MAASSPTPGSGLGTILAAGDRDYLVRNSGEQVKISSIEGDTVAVYFSASWCPPCQRFTP
KLIEAYKELTSHGKSFEVIFVSGDQDEEAFNAYFAKMPWLAVPFSDSEGRKSLDERFEV
NGIPHLVFLDAKTGEVLTDEGVEFVSEYGIEAYPFTAERIDELKEQEKAAKDNQTIHSV
LGTPNRNYVISNTGEKVPIVDLEGKYVGICFVVNGYPPVVEFTPVLAKIYAKLKEVGEK
FEVVAVSLDSDEESFNNSFSSMPWLAIPQGDKMCQKLVSYFELSDLPTLVLIGPDGKTL

SSNIADIINEHGLDAWEGFPFNAEKLEILAEKAKAKAAAQTLESLLVTGDIDFVIGKDG
AKVPVAELVGKTVLLYFSAKWCGPCRAFLPTLVDVYNKVKEKNSDFEIVFISSDRDQSS
FDDFFSGMPWLAVPLEDDRKAYLKKKFKIRGIPSLVAIGPDGKTVNTDAKTSLAVHGAD
AFPFTSERIQELEKKIDEMAKGWPEKVKHELHEHELVLIRRPRPYGCDGCEEMGTSWSY
NCAECDFDLHTKCALGEEKKGDEVNGQDAAAEAAAPAGYVCEGDVCRKA

NTR

>TaNTRA [PHYTOZOME:Traes_6DL_3945FC9B7.2][KEGG:123128174]
MEGSAAAAPLRTRVCIIGSGPAAHTAAIYAARAELKPVLFEGWMANDIAAGGQLTTTTDVENFP
GFPTGIMGIDLMDNCRAQSARFGTNILSETVTEVDFSARPFRVTSDSTTVLADTVVVATGAVAR
RLHFPGSETYWNRGISACAVCDGAAPIFRNKPIAVIGGGDSAMEEGNFLTKYGSQVYIIHRRNT
FRASKIMQARALSNPKIQVVWDSEVVEAYGGAGGGPLAGVKVKNLVTGEVSDLKVSGLFFAIGH
EPATKFLNGQLELHADGYVATKPGSTHTSVEGVFAAGDVQDKKYRQAITAAGSGCMAALDAEHY
LQEVGAQEGKSD

>TaNTRC [PHYTOZOME:Traes_2BS_DEC12328A.2]
MTTTEVENFPGFPDGITGPDLMDKMRKQAERWGAELHQEDVEFIDVKSRPFVIRSSDRE
VKSHSVIIATGATAKRLRLPREEEFWSRGISACAICDGASPLYKGQVLAVVGGGDTATE
EAIYLTKYACHVHLLVRRDQLRASKAMQDRVLNNPNITVHFNTEAVDVVGNTKGQMSGI
QLRRIDTGEEKVLEVKGLFYGIGHTPNSQLLQGQIELDSSGYILVDEGTAKTSVDGVFA
AGDVQDHEWRQAVTAAGSGCIAALSVERYLVSNDLLVEFHQPVREEKKKEIEGKDVEMG
FDITHTKHKGQYALRKLYHGSPRLILVLYTSPTCGPCRTLKPILNKVIDEYDEYVHFVE
IDIEEDPEIAEAAGIMGTPCVQFFKNKEMIRTFSGVKMKKEYREFIESNK

FTRA

>TaFTRA.2.1 [PHYTOZOME:Traes_6DL_1B50CCE2A.2]
MATTAAAMAPPSTSASSVLLLRRLPSPASAAPRCCRLGPPPRLRTARPRVSLTSDVSSS
SDVAEEEAEHAPKVGRRVRVTAPLRVYHVVKAPDLDIQGMEGVIKQYVGVWKGKRITAN
FPFKVEFQLTVDTQPKPVKLFVHLREDEFEYIGDE

FTRB

>TaFTRB.1 [PHYTOZOME:Traes_6DS_8F0F39831.6]
MMSITSTAAAGTPVCRPSVSRGGRRSRCAVRAQADGASSEQKSLEIMRKFSEQYARRSS
TFFCSDKSVTAVVIKGLADHKDQLGAPLCPCRHYDDKAAEAAQGFWNCPCVPMERERKEC
HCMLFLTPDNDFAGEDQAISLEEIKEATSKY
