# Supplementary material for: Thioredoxins as hormone-responsive redox regulators in wheat: transcriptomic and structural insights
Source: Front Plant Sci. 2026 Oct 8;17:1911123. doi: 10.3389/fpls.2026.1911123 (PMC13687799; doi:10.3389/fpls.2026.1911123)
Supplement: Supplementary file 2 [file DataSheet2.pdf]

Supplementary material 2: Primers sequences used for qPCR analysis of wheat *TaTRX* genes

Gene Names	Forward Primer (5'–3')	Reverse Primer (3'-5')
TaNRX1.1	GGGAGATGAGGTGAATGGGC	CCAGCGGGGTACGTGTGC
TaNRX1.2	ACTTCGATCTGCACACCAAGT	CTTCTTCTCCTCACCCAGCG
TaNRX1.3	GAGAGGAGGTGAATGGGCAG	CCAGCTGGGTACGTGTGC
TaTRX-h1.4	GTTGTGGGAGCTGCCAAGG	TTGCACTGTGCTTCGCAATC
TatTRX-L3.1.3	GCCTTCAGAAAATGCCCACA	TTCTCCATCCTTCCACAACTGA
TaCDSP32.1	CCTTCCTCTTCATCAGGGACG	TTGCCGGAGCCGACGTAG
TaACHT4.2	AAACCATTGGTGCGGCAGG	ACCAAGGTCCTCTCTCCGGA
TaACHT6.1	TTGCCAAGTACACCTCACCTG	TTACTCCTCCGGCTGCGTCT
TaACHT6.3	CCATTGCCAAGTACACATCACC	GCCTGAGACGGAGCCGGAG
TaACHT6.4	GGGTCCTGGTGGAGTCCTTC	GGACAAGGACAGGATCACCG
Taβ-actin	TACTCCCTCACAACAACC	GCTCCTGCTCATAATCAAG
