# Supplementary material for: Thioredoxins as hormone-responsive redox regulators in wheat: transcriptomic and structural insights
Source: Front Plant Sci. 2026 Oct 8;17:1911123. doi: 10.3389/fpls.2026.1911123 (PMC13687799; doi:10.3389/fpls.2026.1911123)
Supplement: Supplementary file 3 [file DataSheet3.pdf]

Supplementary material. 3: Predicted docking affinities of TaTRX with phytohormones. Comparison of blind and targeted docking affinities. Blind docking, designated as B, represents an algorithmic search to identify the most favorable binding sites within the whole protein structure for a given ligand. Targeted docking, designated as T, focuses on a selected region of the protein, in this case the region containing the active-site residues previously identified in Table 1. A cutoff value of ≤ −6.5 kcal/mol, based on blind docking, was used to select proteins with the highest predicted binding affinity. The selected sequences are highlighted in green.

Class	Protein Name	Protein ID	ABA (kcal/mol)		IAA (kcal/mol)		MeJA (kcal/mol)	
			B	T	B	T	B	T
ACHT	TaACHT4.1	Traes_4AS_B1D4048C4.1	-6.6	-4.7	-5.9	-4.9	-5.5	-3.9
	TaACHT4.2	Traes_2DS_5A8D0F48E.1	-5.9	-3.8	-6.2	-4	-5.2	-3.7
	TaACHT6.1	Traes_2AL_63BE91CB7.1	-5.7	-4.8	-5.7	-4	-4.8	-3.7
	TaACHT6.2	Traes_2DL_FFF62E69D.1	-5.5	-4.7	-5	-4.5	-4.3	-3.5
	TaACHT6.3	Traes_2BL_D82F44AF2.1	-6.1	-4.5	-6	-4.3	-5.5	-3.8
CDSP32	TaCDSP32.1	Traes_6DL_C6E63ED1C.1	-7.5	-5.2	-7.3	-5.1	-6.3	-4.5
	TaCDSP32.2	Traes_6AL_30768AD47.1	-6.4	-6	-5.4	-5.1	-5.4	-5.2
TRX-h	TaTRX-h1.3	Traes_1AL_11FF51731.1	-5.8	-3.8	-5.3	-4.7	-4.5	-4.4
TRX-L	TaTRX-L3.1.1	Traes_6BL_69E800C48.1	-6.4	-4.7	-6	-4.5	-5.8	-3.7
	TaTRX-L3.1.2	Traes_6DL_3D072DB07.2	-6	-4.8	-5.6	-4.5	-4.7	-4.2
	TaTRX-L3.1.3	Traes_6AL_768707567.2	-6.7	-5.2	-5.8	-4.7	-5.3	-4.5
NRX	TaNRX1.1	Traes_2DL_28DFAC79D.2	-7.1	-5.1	-6.1	-4.8	-6	-4.4
	TaNRX1.2	Traes_2AL_D80447132.1	-6.8	-5	-7.3	-4.6	-6.1	-4.5
	TaNRX1.3	Traes_2BL_34819D129.1	-6.5	-5.8	-7	-5	-5.6	-5.1
